# SM22α^+^ vascular mural cells are essential for vessel stability in tumors and undergo phenotype transition regulated by Notch signaling

**DOI:** 10.1186/s13046-020-01630-x

**Published:** 2020-07-02

**Authors:** Xinxin Zhang, Xianchun Yan, Jing Cao, Ziyan Yang, Xiuli Cao, Yufei Zhang, Liang Liang, Minhua Zheng, Xiaowei Liu, Jian Zhang, Hua Han

**Affiliations:** 1 State Key Laboratory of Cancer Biology, Department of Biochemistry and Molecular Biology, Fourth Military Medical University, Chang-Le Xi Street #169, Xi’an, 710032 China; 2 Department of Respiratory Diseases, Xijing Hospital, Fourth Military Medical University, Chang-Le Xi Street #127, Xi’an, 710032 China; 3 Department of Medical Genetics and Developmental Biology, Fourth Military Medical University, Xi’an, 710032 China; 4 Department of Urology, Xijing Hospital, Fourth Military Medical University, Xi’an, 710032 China

**Keywords:** Tumor vasculature, Vascular mural cells, Vessel smooth muscle cells, vSMC phenotype switch, Notch signaling

## Abstract

**Background:**

Malformation of blood vessels represents a hallmark of cancers, but the role and regulation of vascular mural cells (vMCs), including vascular smooth muscle cells (vSMCs) and pericytes, in tumors has not been fully understood. SM22α has been identified as a marker of vSMCs. This study aims at elucidating the function and regulation of SM22α^+^ mural cells (SM22-MCs) in tumor stroma.

**Methods:**

Gene-modified mice with a SM22α-CreER^T2^ transgene were employed to deplete SM22-MCs or activate/block Notch signaling in these cells. vSMCs from mouse dorsal aorta (vSMCs-DA) were cultured in vitro. RNA-seq was used to compare gene expression profiles. qRT-PCR and western blotting were used to determine gene expression level. Immunofluorescence was used to observe morphological alterations in tumors.

**Results:**

SM22-MCs are essential for stabilizing tumor vasculature. Notch signaling was downregulated in tumor-derived SM22-MCs and vSMCs-DA treated with cancer cell-derived conditioned medium. Notch activation in SM22-MCs normalized tumor vasculature and repressed tumor growth. On the other hand, Notch disruption aggravated abnormal tumor vasculature and promoted growth and metastasis. Gene expression profiling of vSMCs-DA showed that Notch activation enhances their contractile phenotype and suppresses their secretory phenotype, further attenuating the invasion and proliferation of tumor cells. In contrast, Notch blockade in vSMCs-DA mitigated their contractile phenotype while strengthened the secretory phenotype.

**Conclusion:**

SM22-MCs facilitate vessel stability in tumors, and they gain a secretory phenotype and promote tumor malignancy in the absence of Notch signaling.

## Background

Neovascularization is not only a prerequisite of tumor growth, but also initiates or enhances other malignant behaviors of cancer, such as invasion and metastasis [1, 2]. While endothelial cells (ECs) play multidimensional roles in both physiological and pathological vascularization [3, 4], vascular mural cells (vMCs), including vascular smooth muscle cells (vSMCs) and pericytes, are also essential for vessel development and functions [5]. Under physiological conditions, vMCs are fundamental for maintaining vessel structure and regulating vessel contraction/relaxation and other functions [6]. However, tumor vessels are characterized by reduced and/or abnormal vMCs, leading to destabilized tumor vasculature [2]. Moreover, vMCs often lose their anatomical localization in tumors, and switch from a contractile into a secretory/proliferation phenotype, contributing to the cancer-associated fibroblasts (CAFs) repertoire [7–9]. With the secretory/proliferation phenotype, vMCs produce cytokines and chemokines to facilitate proliferation, invasion, and metastasis of tumor cells and an immune-suppressive milieu to strengthen tumor malignancy [7, 10]. Elucidating the precise functions and regulation of vMCs in tumors could provide novel strategies for efficient tumor therapy [11].

Several signaling pathways and transcriptional factors, such as nuclear factor kappa B (NF-κB), have been implicated in vMCs in tumors [10–12]. The Notch signaling pathway, which is composed of Notch ligands (Dll1, 3, and 4, and Jagged 1 and 2), Notch receptors 1–4, transcription factor recombination signal binding protein Jκ (RBPj), and downstream Hes family effectors, plays a critical role in cell fate determination in vascular development and homeostasis [13, 14]. Notch signaling is initiated by γ-secretase-dependent cleavages of Notch receptors, liberating the Notch intracellular domain (NIC) that serves as a transcription factor to transactivate RBPj. The Notch pathway plays an essential role in the development of vSMCs because mutations in Notch-related molecules have been associated with several human genetic diseases involving vSMCs [14, 15]. More recently, Notch signaling has been implicated in vSMC phenotype switch, which is involved in cardiovascular diseases [15]. Blocking Notch signaling leads to CAF activation and promotes CAF and tumor cell expansion [15–17]. However, the exact role of Notch signaling in vMCs in cancer remains unelucidated.

SM22α is a 22 kDa protein that physically associates with cytoskeletal actin filament bundles in contractile vSMCs [18, 19]. Previous studies have shown that SM22α is abundantly expressed in vSMCs and myofibroblasts in tumors [20]. In this study, we show that SM22α^+^ cells are primarily distributed in the perivascular region of tumors and are essential for vessel stability. Moreover, SM22α^+^ vMC (SM22-MC) phenotypes are modified by the tumor microenvironment (TME). We demonstrate that Notch signaling plays a critical role in regulating SM22-MC phenotypes, namely, Notch activation promotes contractile and represses secretory phenotypes in SM22-MCs. Our results provide an insight into the dual roles of SM22-MCs in tumors, and show that intervening with the phenotype of these cells could help cancer therapy.

## Methods

### Human samples

Samples of low differentiation human lung adenocarcinoma biopsies were purchased from Wuhan Servicebio Technology Co., Ltd. (Wuhan, China) (Additional file 2: Table S1).

### Animals

Mice of C57BL/6 background with specific genetic modifications (Additional file 1: Figure S1A) were maintained under specific pathogen-free (SPF) condition. The SM22α-CreER^T2^ mice, which express a tamoxifen-inducible Cre recombinase under the control of SM22 promoter, were kindly provided by Zhu MH [19]. Rosa-Stop^floxed^-NIC mice harbor a murine Notch1 NIC (amino acids 1749–2293) gene followed by Ires-GFP inserted at the Rosa26 locus, and were originally derived from Jackson Laboratory (stock #008159, Bar Harbor, ME). RBPj^floxed^ mice were previously reported [21]. Cre-mediated recombination activates or blocks Notch signaling by ectopically overexpressing NIC or disrupting RBPj, respectively, in these mice [21, 22]. Rosa-Stop^floxed^-tdTomato (stock #007909) mice and Rosa-Stop^floxed^-DTA (stock # 009669) mice, which label or kill specific cell types by Cre-activated expression of tdTomato or diphtheria toxin A chain (DTA), respectively, were also obtained from Jackson Laboratory. The mice were genotyped by PCR, using tail DNA as a template and primers listed in Additional file 2: Table S2. For induction of Cre-mediated recombination, 6- to 8-week-old male mice were intraperitoneally (i.p) injected every day with tamoxifen (50 mg/Kg, Sigma-Aldrich, St. Louis, MO) for 5 consecutive days.

For tumor models, LLC and B16-F10 melanoma cells were inoculated subcutaneously (s.c) on the right back of mice (1 × 10^6^ cells/100 μl PBS) a day after the last tamoxifen injection. Tumors were removed 21 (LLC) or 16 (B16) days post-inoculation. Tumors were weighed and their sizes evaluated using a caliper (tumor size = π × [d2 × D]/6, with d = short diameter and D = long diameter). For metastasis, LLC cells were transfected with lentivirus overexpressing luciferase (GeneChem Technology, Inc., Shanghai, China), following the supplier’s protocol. Mice were inoculated with luciferase-expressing LLC cells. Tumors were surgically removed on day 14 under anesthesia. The mice were maintained 21 days further, and then injected with D-luciferin (150 mg/Kg, Yeasen Biotechnology, Shanghai, China), and sacrificed 8 min later. Lungs were collected and analyzed using a bioluminescence imaging system (IVIS) (Xenogen, Perkin-Elmer, Fremont, CA, USA). To detect CTCs, LLC cells were labeled with GFP using lentivirus (GeneChem). GFP^+^ LLC cells were inoculated as above. Blood was collected 21 days post-inoculation. After erythrolysis using a Red-lysis buffer (Cwbio, Beijing, China), GFP^+^ cells were counted under a fluorescence microscope (BX51, Olympus, Tokyo, Japan).

### Histology

Mice were anesthetized and cardiac-perfused with PBS. Tumors were harvested, sectioned, and routinely stained with hematoxylin and eosin (H&E). For immunofluorescence, tissues were fixed in 4% paraformaldehyde (PFA) at 4 °C for 4 h and then transferred into 30% sucrose overnight until tissues sank. Samples were embedded in optimal cutting temperature (OCT) compound (Sakura Finetek, Inc., Torrance, CA), cryosectioned at 8-μm thickness, and air-dried for 2 h at room temperature. Sections were blocked and permeabilized with 1% bovine serum albumin (BSA) plus 0.5% Triton X-100 in PBS, and then incubated overnight at 4 °C with primary antibodies. After washing, sections were stained with matched secondary antibodies at 37 °C for 1 h. Images were captured with a confocal fluorescence microscope (A1R, Nikon Instruments, Inc., Shanghai, China). Antibodies are listed in Additional file 2: Table S3. Immunohistochemistry was performed in a similar way, except that the secondary antibodies were horseradish peroxidase (HRP)-labeled and the sections were developed using a 3, 3′-diaminobenzidine (DAB) substrate kit (Zhong Shan Jin Qiao Biotech, Beijing, China). After counter-staining with hematoxylin, images were acquired under a microscope. To assess hypoxia, tumor-bearing mice were injected with pimonidazole hydrochloride (PIMO, 60 mg/Kg) 1 h before tumor collection. Cryosections were then immunostained with a Hypoxyprobe-1-Mab1 kit (Hypoxyprobe, Inc., Burlington, MA), following the manufacturer’s instructions. In some experiments, hypoxia was evaluated by Glut1 immunofluorescence. To evaluate vascular perfusion, mice were injected intravenously (i.v) with 100 μl of FITC-conjugated Dextran-2MD (25 mg/ml) (Sigma-Aldrich). Mice were perfused with PBS 15 min later and tumors were dissected and analyzed using immunofluorescence.

Images were quantitatively analyzed with Image-Pro Plus 6.0 or Image J (NIH). In general, over four random high-power fields of each slide were captured, and independently quantified by two investigators in a blinded fashion. Necrosis, hypoxia and hemorrhage were quantified by calculating necrotic, hypoxic and hemorrhagic areas as the percentage of whole tissue areas in each field. Tumor cell proliferation was quantified by counting the number of Ki67^+^ cells as the percentage of total number of Hoechst^+^ cells in each field. Vessel density was assessed by calculating CD31^+^ area as the percentage of whole tissue areas in each field. Mural cell coverage and vessel perfusion were quantified by the ratio of signal density of SM22α, α-SMA, CNN1, SMMHC, or Dextran-2MD to that of CD31 determined by Image-Pro Plus 6.0. The average proximity of SM22α^+^ cells to vessels was measured by determining the radial distribution of SM22α^+^ cells to the CD31^+^ vessels using the Image J software (NIH).

### Flow cytometry assay

For CD31^+^CD45^−^ cells in peripheral blood detection, peripheral blood was collected and erythrocyte were lysed. Cells were then resuspended and stained with anti-CD31-FITC and anti-CD45-APC antibodies. The concentration of CD31^+^CD45^−^ cells in peripheral blood were analyzed using a FACSCanto II flow cytometer (BD Biosciences, San Jose, CA).

For tdTomato^+^ cells isolation, tissues were harvested and digested in 1 mg/ml collagenase I and 10 μg/mL DNase I (Sigma-Aldrich) for 40 min at 37 °C. After passing through a 70-μm tissue strainer, cell suspensions were centrifuged for 3 min at 1300 rpm at 4 °C, followed by erythrolysis. The tdTomato^+^ cells were sorted using a FACSAriaII™ flow cytometer (BD Biosciences) and immediately used for RNA isolation.

### Cell culture and transfection

LLC, B16-F10, and bEnd.3 murine EC cell lines were purchased from American Type Culture Collection (ATCC, Manassas, VA). Cells were cultured in Dulbecco’s modified Eagle’s medium (DMEM), supplemented with 10% fetal calf serum (FCS) and 2 mM L-glutamine (Invitrogen, Carlsbad, CA). The γ-secretase inhibitor (DAPT, Alexis Biochemicals, Lausen, Switzerland) was used at a concentration of 25 μM.

Adenovirus expressing human Notch1 ICD (AdNIC, 5261 ~ 7665 bp from the 1st coding region nucleotide, NM_017617.4) was purchased from Vigene Biosciences (Parklawn, Rockville, MD). To isolate primary vSMCs, the dorsal aorta of mice was minced mechanically and digested in 1 mg/ml collagenase I and 10 μg/ml of DNase I for 30 min at 37 °C. After passing through a 70-μm tissue strainer, cell suspension was centrifuged for 4 min at 1200 rpm at 4 °C and then resuspended in DMEM containing 10% FBS. Cells (vSMCs-DA) were then plated in a culture dish and transduced with AdNIC or control adenovirus (AdCtrl) at MOI = 300, according to the supplier’s procedures. Cells were harvested 24 or 48 h post-viral infection.

To prepare tumor cell-conditioned medium (TCM), tumor cells were plated (5 × 10^6^ cells) and cultured overnight in 10-cm culture dishes, and the medium replaced with 10 ml serum-free medium (SFM) at about 70% confluence. The medium was collected 48 h after the medium change, filtered through a 0.22-μm filter, and centrifugated for 10 min at 12,000 rpm at 4 °C. vSMCs-DA (1 × 10^5^ cells/ml) were treated with TCM for 24 or 48 h, with SFM as a control.

To prepare CM from vSMCs-DA, cells were seeded and cultured overnight in 6-well plates at a density of 5 × 10^5^ cells/well, and then transduced with AdNIC or AdCtrl, or treated with DAPT and DMSO for 48 h. The medium was then replaced with 1.5 ml serum-free medium. The medium was collected 48 h later, and filtered through a 0.22-μm filter, and centrifugated for 10 min at 12,000 rpm at 4 °C before use.

### In vitro cell proliferation, migration, and adhesion assays

Cell proliferation was evaluated by incubation with 50 μmol/L EdU (RiboBio Co., LTD, Guangzhou, China) in complete medium for 2 h and then fixed in 4% PFA at room temperature for 30 min. Cells were stained with Apollo 567 according to the standard procedures. Images were captured with a fluorescence microscope. Cell proliferation was evaluated by the number of EdU^+^ cells in total Hoechst^+^ cells in at least three fields of each stained sample.

For cell migration, AdNIC- or AdCtrl-transduced or DAPT- or DMSO-treated vSMCs-DA were dissociated and cultured in a Transwell chamber (Merck Millipore, Darmstadt, Germany) in complete medium for 24 h. Cells migrating to the lower side of the polycarbonate membrane were stained with crystal violet and evaluated under a microscope. Cell invasion was evaluated in a similar way using a matrigel invasion chamber (Corning, NY), according to the manufacturer’s instructions. Briefly, LLC or B16-F10 cells were seeded in the invasion chambers and CM from vSMCs-DA was added to the lower chambers. After culturing for 24 h, cells were fixed in 4% PFA for 20 min and stained with crystal violet as above. The number of crystal violet^+^ cells per field were counted using Image-Pro Plus 6.0.

To evaluate vSMCs-DA adhesion, cells were incubated with Vybrant® DiI (Thermo Fisher Scientific, Waltham, MA) for 15 min at 37 °C and then washed with serum-free medium for 3 times. The labeled cells were plated in 24-well plates, which were pre-coated with bEnd.3 cells. Non-adherent cells were discarded 2 h later by washing with serum-free medium for 3 times. The number of DiI^+^ adhesion cells per field were observed under a fluorescence microscope and counted using Image-Pro Plus 6.0.

### Collagen gel contraction assay

Collagen gel contraction assay was performed as described [23]. Briefly, vSMCs-DA were resuspended in serum-free medium at 1 × 10^7^/ml, and 10 μl of the cell suspension was mixed with 90 μl of cold neutralized collagen gel solution (Solarbio, Beijing, China) and added to one well of low adhesion 96-well cell contraction plate (Corning). Gels were allowed to solidify for 20 min at 37 °C. After polymerization, 100 μl of medium containing 1% FBS was added to the well. Gels without cells were incubated as negative control. After 24 h, the gels were fixed in 4% PFA for 20 min and captured by a camera. The area of each collagen gel was determined by Image-Pro Plus6.0, and the contraction rate (%) was calculated as (x-n)/n (x = area of each group with cells, n = area of negative groups).

### Enzyme-linked immunosorbent assay (ELISA)

TNFα and Cxcl10 were evaluated using ELISA kits (Thermo Fisher) according to the manufacturer’s instructions. The results were read at 450 nm using a microplate reader (Biotek, Winooski, Vermont).

### RNA sequencing

RNA sequencing and data analyses were conducted by a commercial service (RiboBio). Briefly, vSMCs-DA transfected with AdNIC or AdCtrl for 48 h were subjected to RNA extraction, using the Trizol reagent (Thermo Fisher). The RNA integrity was evaluated using the Agilent 2200 Tape Station (Agilent Technologies, Santa Clara, CA). Ribosomal RNA (rRNA) was removed using the Epicentre Ribo-Zero rRNA Removal Kit (Illumina, San Diego, C). The remaining RNA was fragmented into approximately 200 bp fragments. Subsequently, samples were subjected to first- and second-strand cDNA synthesis, followed by adaptor ligation and enrichment with a low-cycle PCR using Tru Seq® RNA LT/HT Sample Prep Kit (Illumina). The purified library products were evaluated using the Agilent 2200 Tape Station and Qubit® 2.0 (Life Technologies, Foster City, CA) and then diluted to 10 pM for cluster generation in situ on the HiSeq3000 pair-end flow cell, followed by sequencing (2 × 150 bp) on HiSeq3000. Bioinformatic analyses were performed using the OmicShare tools at www.omicshare.com/tools or TBtools at github.com/CJ-Chen/TBtools/releases.

### qRT-PCR

Total RNA was isolated and precipitated using TRIzol. cDNA was synthesized using a reverse transcription kit (Takara Biotechnology, Dalian, China). Real-time PCR was performed using a SYBR Premix Ex Taq Kit (Takara Biotechnology) and ABI PRISM7500 real-time PCR system (Life Technologies), with β-actin as an internal control. Primers are listed in Additional file 2: Table S2.

### Western blotting

Cells were lysed in the RIPA buffer (Beyotime, Shanghai, China), containing 10 mM phenylmethanesulfonyl fluoride (PMSF), and the protein concentration determined using a BCA protein assay kit (Thermo Fisher). To detect the protein level of nuclear p65, the nuclear extraction was prepared using an extraction kit (Beyotime) according to the manufacturer’s instruction. Total cell lysates and nuclear proteins were then separated by sodium dodecyl sulfate-polyacrylamide gel electrophoresis (SDS-PAGE), transferred onto polyvinylidene fluoride (PVDF) membranes, probed with primary antibodies, followed by HRP-conjugated secondary antibodies. β-actin was used as a loading control. HRP-based detection was done using an enhanced chemiluminescence (ECL) system (Clinx Science Instruments, Shanghai, China). Antibodies are listed in Additional file 2: Table S3.

### Statistical analyses

Statistical analyses were performed with Image-Pro Plus 6.0, Image J (NIH) and GraphPad Prism 8.0 software. Data are expressed as means ± SD. Statistical significance was calculated using Student’s *t*-test. *P* < 0.05 was considered significant.

## Results

### SM22-MCs are required for tumor vasculature maintenance

**Fig. 1:**
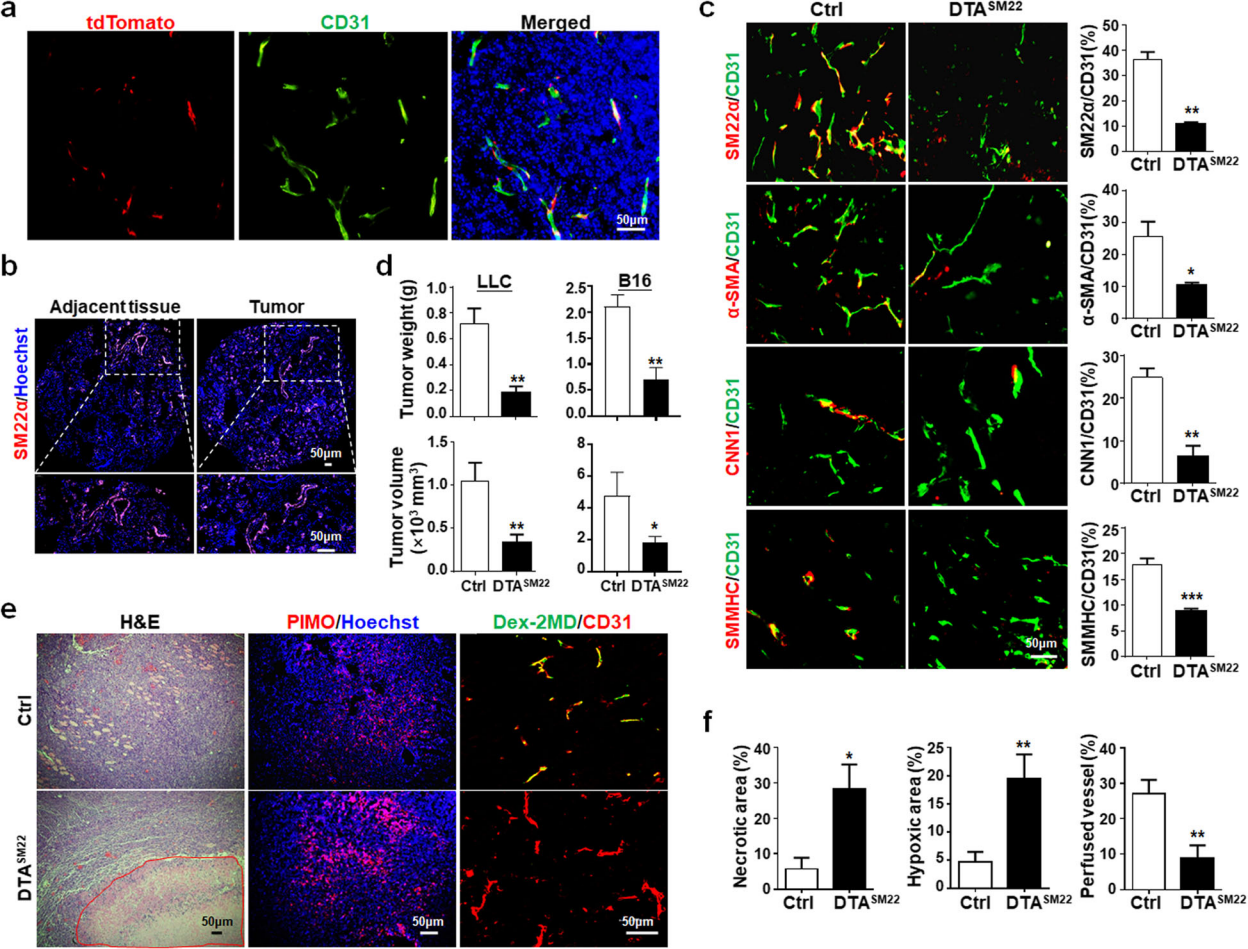
Depletion of SM22α^+^ vMCs promotes tumor growth accompanied by aggravated tumor vessel abnormality. **a** LLC cells were inoculated tdTomato^SM22^ mice for 21 days, and tumor sections were immunostained with CD31. Images represent 3 independent experiments. **b** Human lung cancer and adjacent tissues were immunostained with SM22α. The experiment was repeated 6 times and representative images with different magnifications are shown. **c** LLC cells were inoculated in DTA^SM22^ and Ctrl mice for 21 days. Tumor sections were coimmunostained with CD31 plus SM22α, α-SMA, CNN1, or SMMHC. The percentages of SM22α/CD31, α-SMA/CD31, CNN1/CD31, and SMMHC/CD31 were quantitatively compared (*n* = 4). **d** LLC or B16 cells were inoculated in DTA^SM22^ and Ctrl mice. The weight and volume of tumors were compared between the DTA^SM22^ and Ctrl groups (*n* = 7 for DTA^SM22^ and *n* = 6 for Ctrl in LLC; *n* = 3 for B16). **e-f** Tumor sections from (**c**) were stained with H&E or PIMO. The red line marks the necrotic area after H&E staining. For the rightmost samples, tumor-bearing mice were injected i.v with FITC-conjugated Dextran-2MD 15 min before sacrifice. Tumor tissues were immunostained with CD31. The necrotic and hypoxic areas and perfused vessels (CD31^+^Dex-2MD^+^) were quantitatively compared between the DTA^SM22^ and Ctrl mice (*n* = 5). Bars = means ± SD. *, *P* < 0.05; **, *P* < 0.01

SM22α is normally expressed in vSMCs and detected in tumor stroma [20]. To clarify SM22α^+^ cell distribution in tumors, we crossed SM22α-CreER^T2^ and Rosa-Stop^floxed^-tdTomato mice [19] (Additional file 1: Figure S1A, *a*). The double transgenic mice (tdTomato^SM22^) were induced with tamoxifen, and Lewis lung carcinoma (LLC) cells were inoculated. Immunofluorescence of tumor sections confirmed that tdTomato co-stained with SM22α, suggesting that SM22α marks vSMCs in tumors (Additional file 1: Figure S1B). Moreover, immunostaining of tumor sections showed that some SM22α^+^ cells (tdTomato^+^) colocalized with CD31, but a considerable fraction of tdTomato^+^ cells were distributed in tumor stroma (Fig. 1a). In human lung cancer biopsies, immunofluorescence showed that SM22α^+^ cells were primarily localized in the perivascular region (Fig. 1b). These results suggested that SM22-MCs are present in both the perivascular regions and stroma in tumors.

To functionally clarify the role of SM22-MCs in tumors, we crossed SM22α-CreER^T2^ with Rosa-Stop^floxed^-DTA mice to obtain DTA^SM22^ mice (Additional file 1: Figure S1A, *b*). Vessel density and perfusion were not altered significantly in organs of DTA^SM22^ mice after tamoxifen induction (Additional file 1: Figure S1C). Tamoxifen administration substantially reduced SM22α^+^ cells as well as α-SMA-, CNN1-, and SMMHC-positive stains in tumors of DTA^SM22^ mice (Fig. 1c). We found that the growth of LLC and B16 tumors was significantly suppressed by genetic depletion of SM22-MCs in mice, as shown by significantly decreased tumor weight and volume in DTA^SM22^ mice, as compared with the control (Fig. 1d). Consistently, H&E staining showed remarkably increased necrosis in LLC and B16 tumors in DTA^SM22^ mice. Tumor hypoxia was obviously enhanced, suggesting aggravated vessel dysfunction. Indeed, vessel perfusion was significantly damaged after the genetic depletion of SM22-MCs, although the vessel density in tumors did not change significantly (Fig. 1e, f; Additional file 1: Figure S1D; Figure S2A-C). Taken together, SM22-MCs are required for maintaining tumor vasculature.

### Notch activation in SM22-MCs reduces tumor growth and normalizes vasculature

Notch signaling has been implicated in the development and homeostasis in vSMCs, under physiological and pathological conditions [14–17]. Next, we asked whether Notch signaling could play a role in SM22-MCs in tumors. We cultured primary murine vSMCs from the dorsal aorta (vSMCs-DA), which were strongly positive for SM22α and α-SMA, but negative for the endothelial marker CD31, as shown by immunofluorescence (Additional file 1: Figure S3A). vSMCs-DA were cultured with conditional medium from LLC or B16 tumor cells (TCM), with serum-free medium (SFM) as a control. Reverse transcription-quantitative polymerase chain reaction (qRT-PCR) showed that the mRNA level of Notch-associated genes was downregulated in vSMCs-DA treated with TCM (Additional file 1: Figure S3B, C). Western blotting confirmed that Jag1 and Hes1 protein levels decreased prominently (Additional file 1: Figure S3D). Then, tdTomato^+^ cells were sorted from LLC tumors and adjacent lung tissues of tdTomato^SM22^ mice using a fluorescence-activated cell sortor (FACS). qRT-PCR showed that Hey1 and Hey2 expression decreased markedly in tdTomato^+^ SM22-MCs from tumors, compared with those from lung tissues (Additional file 1: Figure S3E). These results suggested that TME-derived cues repressed Notch signal activation in SM22-MCs.

**Fig. 2:**
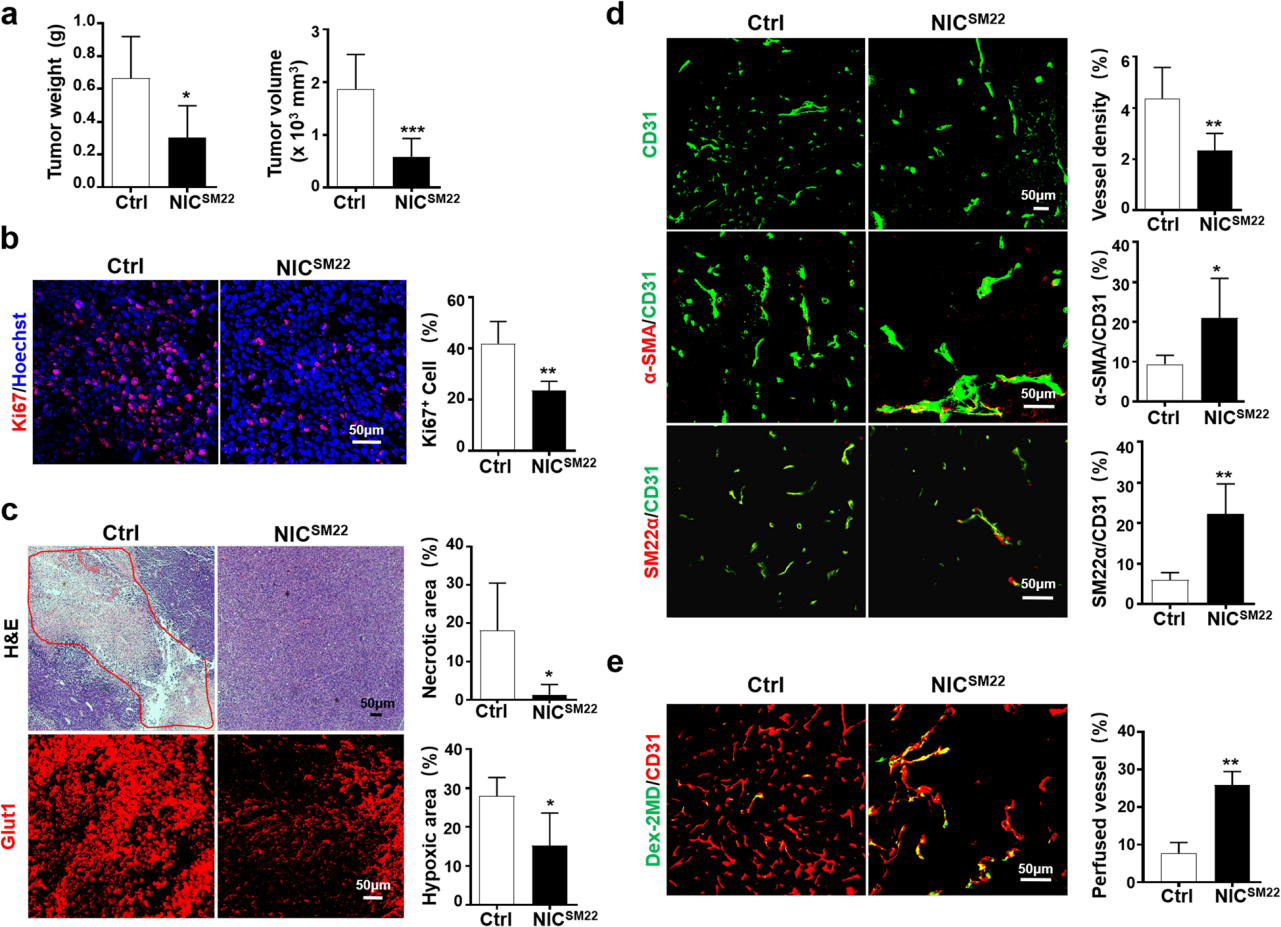
Notch activation in SM22-MCs suppresses tumor growth and normalizes tumor vessels. **a** NIC^SM22^ and Ctrl mice were inoculated with LLC cells. Tumor weight and volume were determined 21 days later (*n* = 7 for NIC^SM22^ and *n* = 6 for Ctrl). **b** Tumor sections were immunostained with Ki67. The percentages of Ki67^+^ cells were compared (*n* = 5). **c** Tumor sections were stained with H&E (*n* = 5) or Glut1 immunofluorescence (*n* = 4). The necrotic (marked by red lines) and hypoxic (Glut1^+^) areas were evaluated and compared between the NIC^SM22^ and Ctrl mice. **d** Tumor sections were immunostained with CD31, α-SMA, and SM22α. Vessel density and vMC coverage were quantitatively compared between the NIC^SM22^ and Ctrl mice (*n* = 6 for CD31; *n* = 5 for CD31 plus α-SMA; and *n* = 4 for CD31 plus SM22α). **e** Mice bearing LLC tumors were injected i.v with FITC-conjugated Dextran-2MD 15 min before sacrifice. Tumor tissues were then immunostained with CD31. Perfused vessels (CD31^+^Dex-2MD^+^) were quantitatively determined (*n* = 4). Bars = means ± SD. *, *P* < 0.05; **, *P* < 0.01; and ***, *P* < 0.001

To evaluate the role of Notch signaling in SM22-MCs in tumors, we generated SM22α-specific Notch activation mice, in which ectopic NIC expression is activated by SM22α-CreER^T2^ upon tamoxifen administration [22] (NIC^SM22^, Additional file 1: Figure S1A, *c*). qRT-PCR analysis of vSMCs-DA indicated that upon tamoxifen induction, the mRNA level of NIC and Hes1 increased in NIC^SM22^ mice (Additional file 1: Figure S3F), suggesting that Notch signaling was activated. LLC cells were inoculated in NIC^SM22^ and control mice. The growth of LLC tumors was apparently slower in NIC^SM22^ mice than in the control (Fig. 2a). Ki67 immunostaining showed that tumor cell proliferation decreased in NIC^SM22^ mice (Fig. 2b). Tumor necrosis and hypoxia were markedly reduced in LLC tumors from NIC^SM22^ mice, as shown by H&E staining and Glut1 immunofluorescence [24, 25], respectively (Fig. 2c). Immunofluorescence also showed that Notch activation in SM22-MCs reduced vessel density and increased the mural cell coverage of tumor vasculature (Fig. 2d). Vessel perfusion was improved significantly in tumors of NIC^SM22^ mice (Fig. 2e). These results suggested that Notch activation, which is repressed in TME, in SM22-MCs promotes the structural and functional normalization of tumor vessels to suppress tumor growth.

### Notch blockade by RBPj disruption in SM22-MCs promotes tumor growth and metastasis

**Fig. 3:**
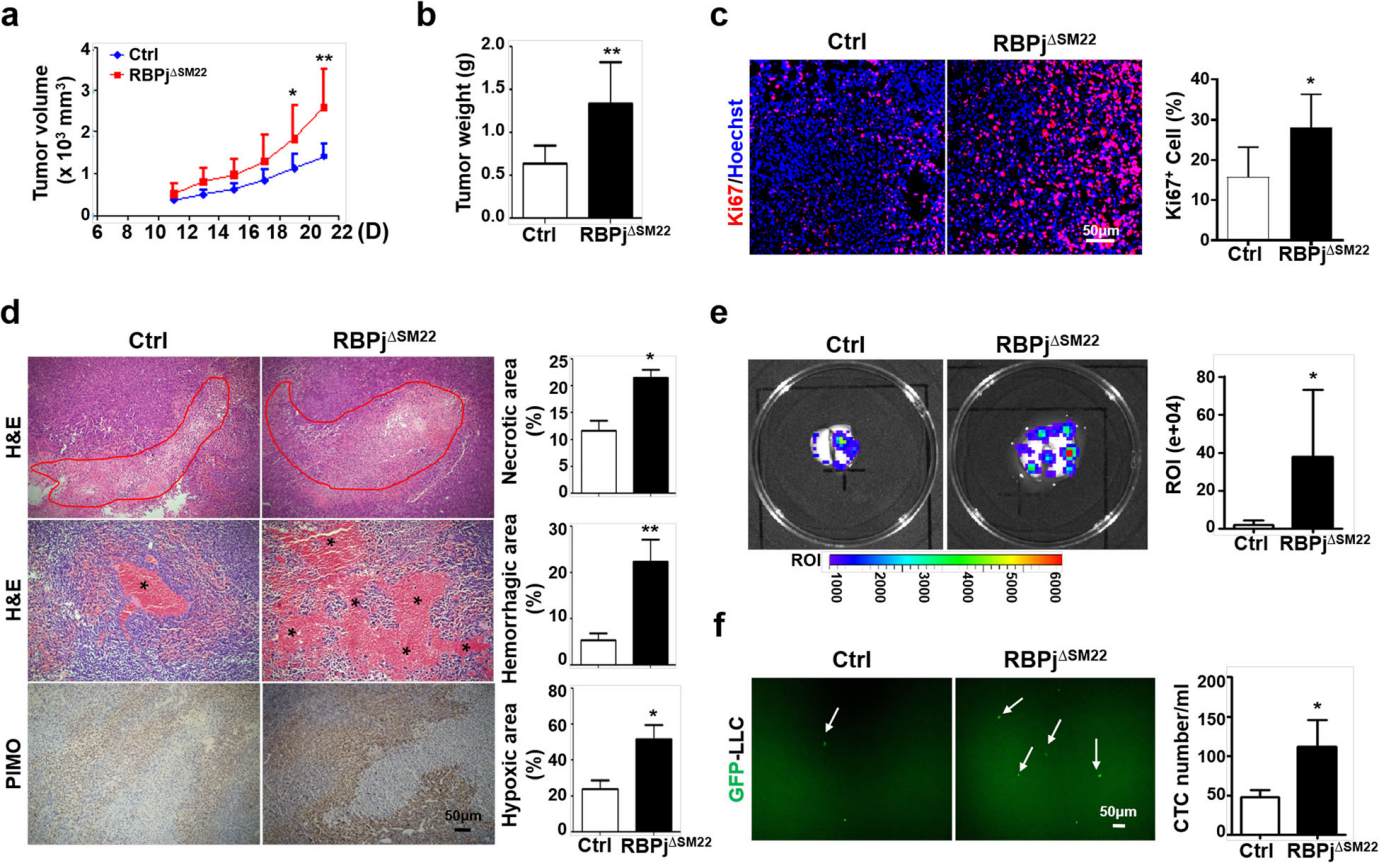
RBPj deficiency in SM22-MCs promotes tumor malignancy. **a-b** RBPj^∆SM22^ and Ctrl mice were inoculated with LLC cells for 21 days. Tumor volume was monitored every other day from day 11. Tumor weight was measured on day 21 (*n* = 5). **c** Tumor sections were stained with Ki67. The percentages of Ki67^+^ cells were quantitatively compared between the RBPj^∆SM22^ and Ctrl mice (*n* = 5). **d** Tumor sections were stained with H&E or PIMO. The necrotic (marked by red lines), hemorrhagic (marked by stars), and hypoxic areas (PIMO^+^) were quantitatively compared between the RBPj^∆SM22^ and Ctrl mice (*n* = 5). **e-f** LLC cells were transfected with lenti-luciferase (**e**) or lenti-GFP (**f**) and inoculated in the RBPj^∆SM22^ and Ctrl mice. Tumors were removed on day 14 and the mice were maintained for 21 more days. Lung metastasis was evaluated under an in vivo imaging system (**e**) (*n* = 6 for Ctrl and *n* = 5 for RBPj^∆SM22^). Circulating GFP^+^ LLC cells in the blood (white arrows) was determined using a fluorescence microscope and quantitatively compared (**f**) (*n* = 4 for Ctrl, *n* = 3 for RBPj^∆SM22^). Bars = means ± SD. *, *P* < 0.05; and **, *P* < 0.01

To further determine the role of Notch signaling in SM22-MCs in tumors, we generated inducible vSMC-specific RBPj knockout mice (RBPj^∆SM22^) by crossing the SM22α-CreER^T2^ and RBPj^floxed^ mice [21] (Additional file 1: Figure S1A, *d*). PCR analysis indicated that tamoxifen induction significantly deleted the floxed RBPj exon (exon 6) in vSMCs-DA of RBPj^floxed^ mice [21], and qRT-PCR showed that the mRNA level of RBPj (detected with primers within and outside the deleted exon) and Hes1 decreased in RBPj^∆SM22^ mice (Additional file 1: Figure S4A, B), suggesting that Notch signaling was suppressed. The growth of LLC tumors accelerated significantly in RBPj^∆SM22^ mice (Fig. 3a, b). Tumor cell proliferation increased, accompanied by increased necrosis, hemorrhage, and hypoxia in tumor tissue in the RBPj^∆SM22^ mice (Fig. 3c, d). Similar effects were noticed in B16 melanoma in RBPj^∆SM22^ mice (Additional file 1: Figure S4C, D).

Evaluation of tumor metastasis revealed that RBPj deficiency in SM22-MCs promoted lung metastasis of subcutaneous LLC tumors (Fig. 3e). Consistently, circulating tumor cells (CTCs) increased significantly in RBPj^∆SM22^ mice (Fig. 3f). These results indicated that Notch blockade in SM22-MCs promotes tumor malignancy, likely via aggravating dysfunctional tumor vasculature [26].

### RBPj deficiency in SM22-MCs leads to disrupted tumor vasculature

**Fig. 4:**
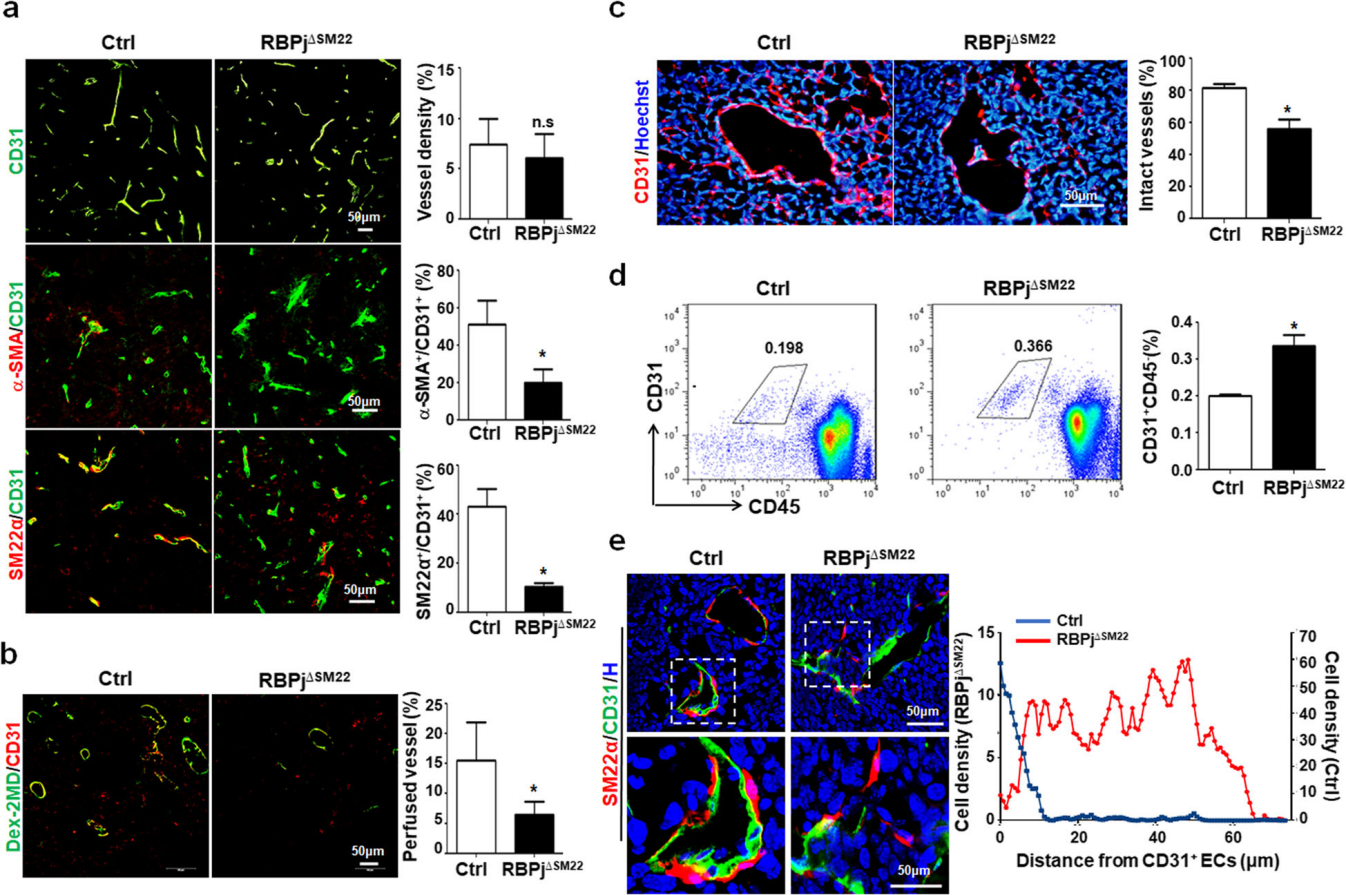
RBPj deficiency in SM22-MCs results in disordered tumor vasculature with poor function. **a** RBPj^∆SM22^ and Ctrl mice were inoculated with LLC cells for 21 days. Tumor sections were stained with CD31 and CD31 plus α-SMA or SM22α. Tumor vessel density and vMC coverage were quantitatively compared (*n* = 5). **b** Vessel perfusion was determined by injection of Dextran-2MD, followed by immunostaining. The percentages of perfused vessels were compared between the RBPj^∆SM22^ and Ctrl mice (*n* = 5). **c** Tumor sections were immunostained with CD31. The intact vessels, which were determined by lumens with continuous CD31^+^ ECs, were determined and compared (*n* = 5). **d** RBPj^∆SM22^ and Ctrl mice were inoculated with LLC cells. The CD31^+^CD45^−^ ECs in blood were determined by FACS on day 21 and compared between the RBPj^∆SM22^ and Ctrl mice (*n* = 5). **e** Tumor sections were immunostained with CD31 and SM22α. The distance between SM22-MCs and CD31^+^ ECs was determined and quantitatively compared between the RBPj^∆SM22^ and Ctrl mice. Bars = means ± SD. *, *P* < 0.05 and n.s, not significant

We then examined tumor vasculature in RBPj^∆SM22^ mice by immunostaining. The results showed that while vessel density did not change in the tumors in RBPj^∆SM22^ mice, mural cell coverage decreased markedly as shown by co-staining CD31 with α-SMA and SM22α (Fig. 4a). In B16 melanoma in RBPj^∆SM22^ mice, vessel density increased and mural cell coverage decreased (Additional file 1: Figure S4E, F). Tumor vessel perfusion in the RBPj^∆SM22^ mice decreased as compared with the Ctrl (Fig. 4b). We further looked at vessel integrity and found that vessels with intact EC coverage in the lumen side decreased significantly in RBPj^∆SM22^ mice (Fig. 4c). Meanwhile, FACS analysis showed that more CD31^+^ cells were detected in peripheral blood in tumor-bearing RBPj^∆SM22^ mice, as compared with the Ctrl (Fig. 4d), likely attributable to detachment of ECs from microvessels [27]. Together, these results suggested that Notch blockade in SM22-MCs aggravates abnormalities in tumor vasculature, leading to enhanced tumor malignancy.

### Notch activation reinforces the contractile phenotype in SM22-MCs

**Fig. 5:**
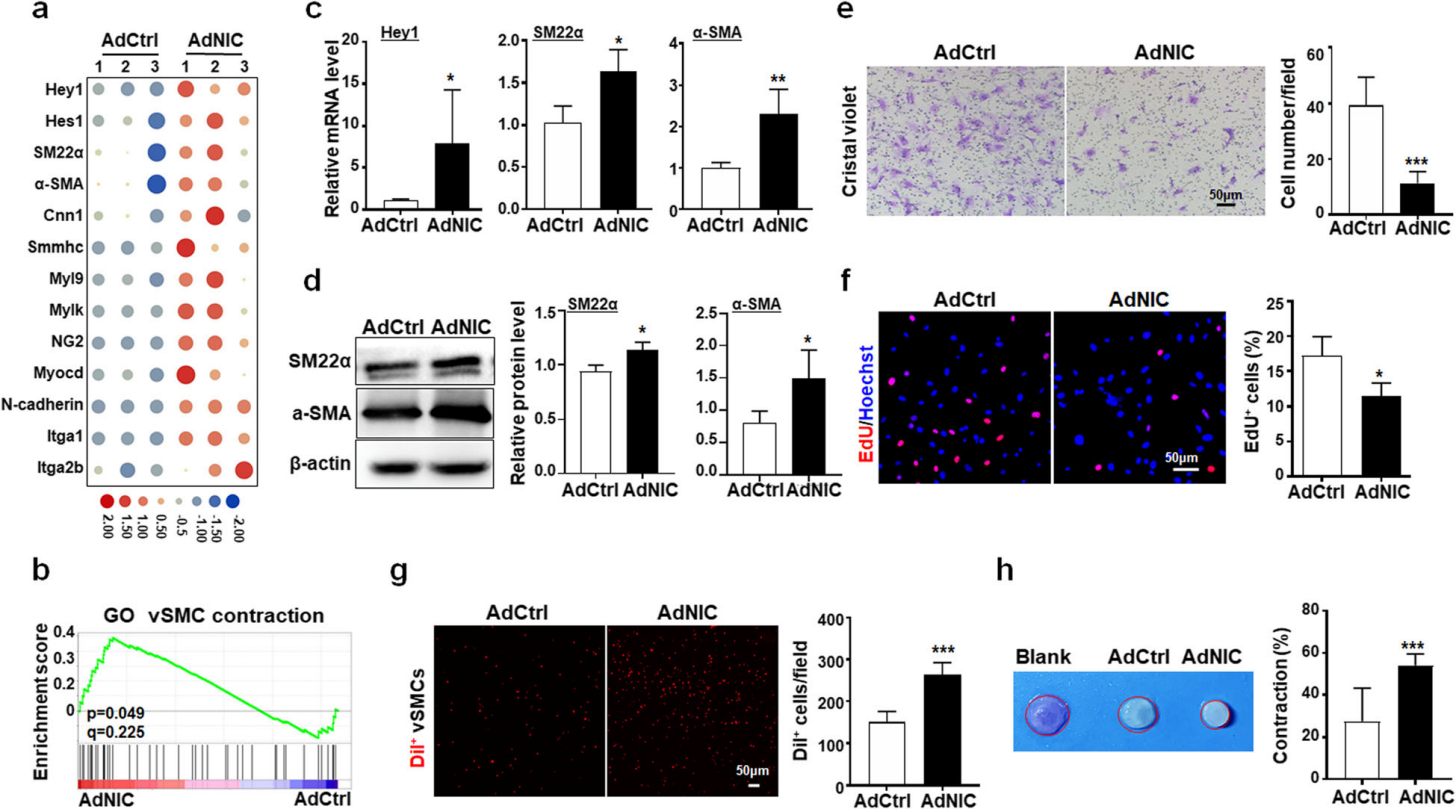
Notch activation promotes the contractile phenotype in vSMCs. **a-b** vSMCs-DA were transduced with AdCtrl or AdNIC for 48 h and subjected to RNA sequencing. The expression of vSMC-associated genes is shown by a heatmap (**a**). The enrichment score of vSMC contraction-associated genes was analyzed using GSEA (**b**). **c-d** vSMCs-DA were transduced with AdCtrl or AdNIC for 48 h. The expression of SM22α and α-SMA was determined using qRT-PCR (**c**) and western blotting (**d**) and quantitatively compared (*n* = 5). **e** AdCtrl- or AdNIC-transduced vSMCs-DA were plated in Transwell chambers and incubated for 24 h. The migration of cells to the lower chamber was evaluated by crystal violet staining and quantitatively compared (*n* = 5). **f** AdCtrl- or AdNIC-transduced vSMCs-DA were incorporated with EdU for 2 h. The percentage of EdU^+^ proliferating cells was determined under a fluorescence microscope (*n* = 3). **g** AdCtrl- or AdNIC-transduced vSMCs-DA were labeled with Dil and cocultured with bEnd.3 ECs for 2 h. After washing, vSMCs-DA adhering to ECs were determined under a fluorescence microscope and quantitatively compared (*n* = 5). **h** AdCtrl- or AdNIC-transduced vSMCs-DA cells were subjected to collagen gel contraction assay (*n* = 5). Relative contraction was compared. Bars = means ± SD. *, P < 0.05; **, *P* < 0.01; and ***, *P* < 0.001

We noticed that in the RBPj^∆SM22^ mice, more SM22α^+^ cells appeared to be away from the vessels, as shown by quantitatively comparing the distance between CD31^+^ ECs and SM22-MCs (Fig. 4e), suggesting that Notch signaling might be involved in the phenotypic transition in these cells, which could be a consequence of TME-derived stimulation (Additional file 1: Figure S5A-C). To explore the potential influence of Notch modulation on SM22-MCs, we ectopically activated Notch signaling in primary vSMCs-DA by overexpressing NIC with adenovirus, and compared the gene expression profile with the control using RNA-seq (Additional file 1: Figure S6A). Bioinformatic analyses showed that Notch activation led to enhanced expression of contractile phenotype-related genes in vSMCs-DA (Fig. 5a, b). qRT-PCR and western blotting confirmed the upregulation of SM22α and α-SMA at mRNA and protein levels, respectively, in vSMCs-DA upon Notch activation (Fig. 5c, d). Moreover, Notch activation resulted in reduced proliferation and migration in vSMCs-DA (Fig. 5e, f). We tested the adhesion of vSMCs-DA to ECs and contraction ability of vSMCs-DA. The result showed that Notch activation enhanced adhesion between ECs and vSMCs-DA and improved their contraction ability (Fig. 5g, h). These results suggested that Notch activation reinforces the contractile phenotype in SM22-MCs.

### Notch activation represses the secretory phenotype in SM22-MCs

**Fig. 6:**
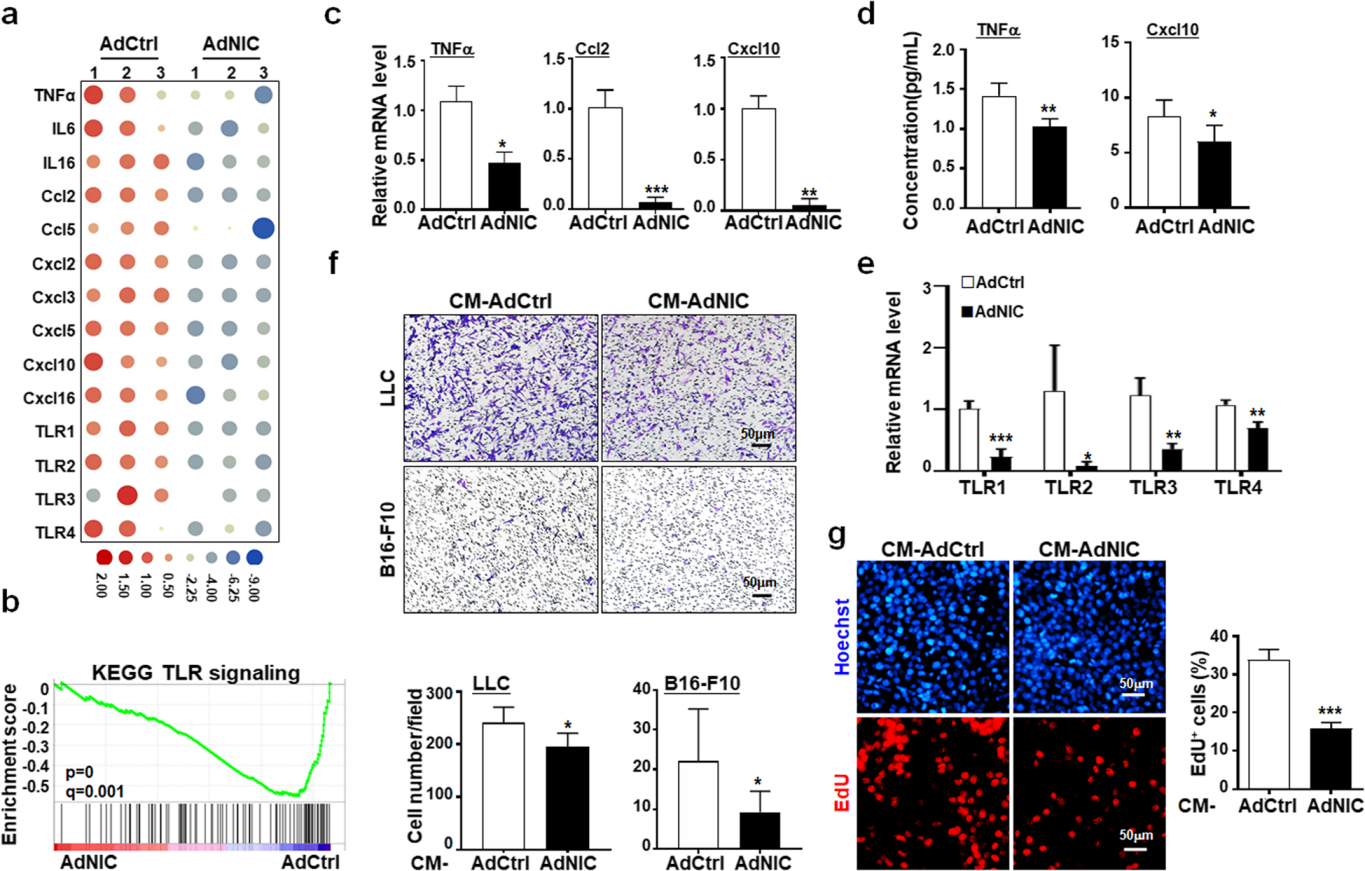
Notch activation alleviates the secretory phenotype in vSMCs. **a** RNA-seq data of AdCtrl- or AdNIC-transduced vSMCs-DA were analyzed for the expression of secretory phenotype-related genes and shown as a heatmap. **b** The expression of secretory phenotype-associated genes was analyzed by GSEA between the AdNIC and AdCtrl groups. **c** vSMCs-DA were transduced with AdCtrl or AdNIC for 48 h. The expression of secretory phenotype-associated genes was determined by qRT-PCR (*n* = 5). **d** vSMCs-DA were treated as in (**c**). The concentration of TNFα and Cxcl10 in the supernatant was determined using ELISA (*n* = 5). **e** Expression of TLR1, TLR2, TLR3 and TLR4 was evaluated using qRT-PCR (*n* = 5). **f** LLC and B16-F10 cells were plated in matrigel-coated Transwell chambers and cultured with CM derived from vSMCs-DA pre-transduced with AdNIC or AdCtrl. The invasion of LLC and B16-F10 cells was determined 24 h later by crystal violet staining (*n* = 5). **g** LLC cells were cultured with CM derived from vSMCs-DA pre-transduced with AdNIC or AdCtrl. The proliferation was evaluated 24 h later using the EdU incorporation assay (*n* = 5). Bars = means ± SD. *, *P* < 0.05; **, *P* < 0.01; and ***, *P*7 < 0.001

Gene ontology (GO) analysis suggested that the AdNIC-transfected vSMCs-DA displayed lowered cytokine secretion and response, suggesting Notch activation likely suppressed the secretory phenotype of SM22-MCs [28–30] (Additional file 1: Figure S6B, C). Indeed, heatmap and GSEA showed that the expression of inflammatory cytokines, chemokines, and toll-like receptors (TLRs) decreased obviously in AdNIC-transfected vSMCs-DA (Fig. 6a, b). qRT-PCR confirmed the downregulation of TNFα, Ccl2, Cxcl10, and TLRs, while ELISA demonstrated that the secretion of TNFα and Cxcl10 decreased in the supernatant from AdNIC-transfected vSMCs-DA (Fig. 6c-e). To evaluate the functional consequence of Notch activation in vSMCs-DA, we collected culture supernatants (CM) from AdNIC- or AdCtrl-infected vSMCs-DA, and treated LLC or B16 tumor cells with these CM preparations. The results revealed that CM from AdNIC-transduced vSMCs-DA could suppress LLC and B16 tumor cell invasion and proliferation, compared with the AdCtrl group (Fig. 6f, g). These data suggested that Notch activation strengthens the contractile phenotype in vSMCs, leading to attenuated tumor malignancy.

### Pharmaceutical notch blockade subverts SM22-MC phenotypes

**Fig. 7:**
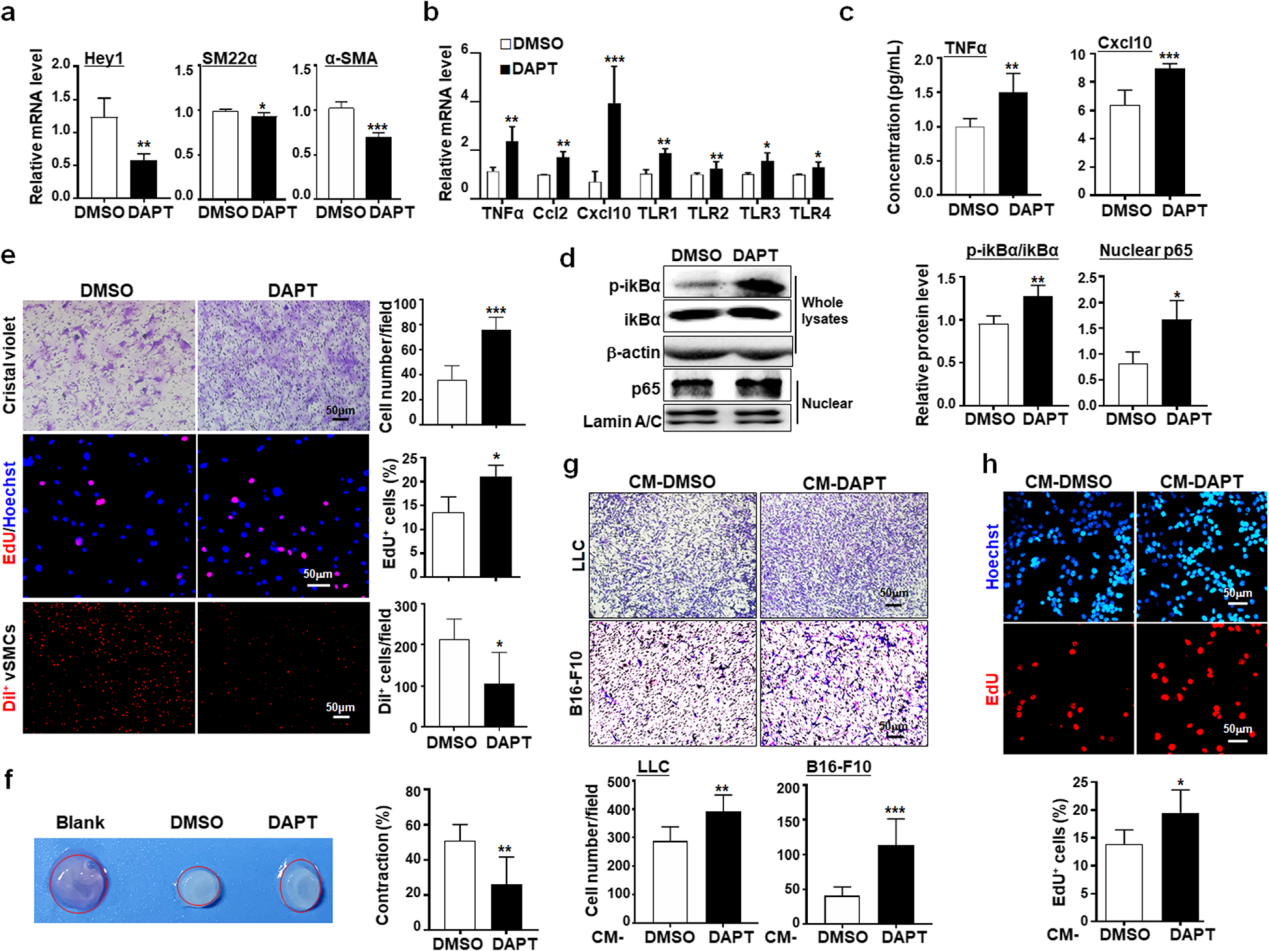
Notch signal blockade abrogates the contractile phenotype and promotes the secretory phenotype in vSMCs. **a-c** vSMCs-DA were treated with DMSO or DAPT for 48 h. The mRNA level of Hey1 and contractile-related genes (**a**) and secretory-related genes (**b**) was determined using qRT-PCR. The concentration of TNFα and Cxcl10 in the supernatant was measured using ELISA (**c**) (*n* = 5). **d** vSMCs-DA were treated with DMSO or DAPT for 48 h. The protein level of total and phosphorylated IκBα and nuclear p65 was determined using western blotting (*n* = 3), with lamin A/C as an internal control. **e** vSMCs-DA were treated with DMSO or DAPT for 48 h. Cell migration was evaluated by Transwell assay (top) (*n* = 5). Cell proliferation was determined using EdU incorporation (middle) (*n* = 3). For adhesion, cells were labeled with Dil and incubated with bEnd.3 cells for 2 h, and evaluated after washing (*n* = 5). **f** vSMCs-DA cells were treated with DMSO or DAPT for 48 h, followed by collagen gel contraction assay in the presence of DMSO or DAPT, respectively (*n* = 5). **g** vSMCs-DA were treated with DMSO or DAPT for 48 h, followed by culturing with normal medium for 48 h. CM was collected and used to culture LLC or B16-F10 cells in a Transwell system for the invasion assay. The invasion of tumor cells was evaluated after 24 h using crystal violet staining (*n* = 7 for LLC; *n* = 10 for B16-F10). **h** LLC cells were treated with vSMCs-derived CM as in (**g**). Cell proliferation was evaluated using the EdU incorporation assay (*n* = 5). Bars = means ± SD. *, *P* < 0.05; **, *P* < 0.01; and ***, *P* < 0.001

We then considered the effect of Notch blockade on the phenotype of SM22-MCs. Fortunately, transcriptomic data of Jagged1 knockout (Jag1-KO) vSMCs isolated from embryonic tissues (E14.5) embryos or immortalized adult vSMCs-DA treated with DAPT has been deposited in a public database [31] (GSE60643). Analyses of these data showed that in embryonic Jag1-KO vSMCs, contractile phenotype-related genes were downregulated, while secretory phenotype-related genes were upregulated, although these changes appeared not significant in DAPT-treated immortalized adult vSMCs (Additional file 1: Figure S7A-F). We then treated vSMCs-DA with DAPT or DMSO. qRT-PCR showed that Hey1, SM22α, and α-SMA expression decreased, while TNFα, Ccl2, Cxcl10, and TLRs were upregulated in DAPT-treated vSMCs-DA (Fig. 7a, b). ELISA also demonstrated that TNFα and Cxcl10 increased significantly in DAPT-treated vSMCs-DA supernatants (Fig. 7c). The NF-κB signaling was activated as shown by the increased level of phosphorylated IκB and nuclear p65 (Fig. 7d). Consistently, Notch blockade by DAPT in vSMCs-DA increased cell proliferation and migration, and reduced adhesion to ECs and the contractibility of vSMCs-DA (Fig. 7e, f). Furthermore, we collected CM from vSMCs-DA after DAPT treatment, and cultured LLC or B16 tumor cells with the CM. The result showed that CM from DAPT-treated vSMCs-DA increased invasion and proliferation of tumor cells (Fig. 7g, h). Together, these results suggested that Notch blockade attenuates the contractile and promotes secretory phenotype in SM22-MCs, which potentially enhances tumor malignancy.

## Discussion

The multi-dimensional roles of stromal cells in tumor malignancy have been appreciated for decades [8, 10]. But, so far controversial results have been reported depending on tumor types and experimental settings. Depletion of CAFs or pericytes induces immunosuppression and hypoxia-associated epithelial-to-mesenchymal transition (EMT), respectively, leading to aggravated malignancy [9, 32]. Xian et al. have also demonstrated that pericytes limit tumor cell metastasis [33]. A more recent report by Tian et al. identified a group of genes associated with good prognosis (GPAGs) in human cancers, many of which are related to vMCs [34]. These findings collectively suggest that stromal cells play a suppressive role in tumor progression. However, on the other hand, many studies have also indicated that tumor stromal cells support tumor growth and exacerbate malignancy [35]. The discrepant functions of stromal cells in tumors could at least partially be attributed to the different subsets of tumor stromal cells, which are supposed to originate from tissue-resident fibroblasts, vMCs, blood-derived cells, and/or tumor cells after EMT [35, 36]. Moreover, phenotypic plasticity of stromal cells could further complicate the situation [8, 10]. In the current study, we have focused on a subset of tumor stromal cells, SM22-MCs. Because SM22α is reportedly expressed by vSMCs with relative specificity [18–20], SM22-MCs mostly represent vessel-derived tumor stromal cells, although we could not provide definite evidence for this opinion. Functionally, SM22-MCs may regulate vascular structure and response to extracellular signals [37]. In our study, genetic depletion of SM22-MCs promotes tumor malignancy, accompanied by exacerbated vessel anomalies, demonstrating that SM22-MCs are essential for vessel stability.

However, the vessel-stabilizing role of SM22-MCs appears to depend on TME. Indeed, vSMCs are highly plastic under various pathological conditions [15, 38]. Our data have also shown that in response to diffusible factors from tumor cells, like other CAF types [35], vSMCs lose their contractile phenotype and acquire a secretory phenotype, likely dampening their vessel-stabilizing capacity and induces tumor cell proliferation, migration, and invasion. Because this phenotypic transition of vSMCs is accompanied by alterations in Notch signaling, which regulates vSMCs phenotypic transition in other context [14, 15], we tried to modulate the phenotypic transition of SM22-MCs in tumors by genetic modification of Notch signaling, specifically in SM22α^+^ cells. Our results have shown that genetic activation of Notch signaling in SM22-MCs results in stabilized tumor vasculature, accompanied by enhanced contractile phenotype and dampened secretory phenotype, while blockade of Notch signaling by conditional knockout of RBPj or pharmaceutical inhibition of γ-secretase exhibits opposite effects. Jag1-KO embryonic vSMCs also show downregulated contractile phenotype-related genes and upregulated secretory phenotype-related genes, likely reflecting the conservation of Notch signaling. These results strongly suggest that in TME, SM22-MCs are likely to be deprived of their vessel-stabilizing ability due to phenotypic transition, which is controlled by Notch signaling and tends to promote tumor growth and metastasis. Of note, SM22-MCs could migrate into tumor stroma, which is likely a result of phenotypic transition, to promote proliferation and invasion of tumor cells and recruit immune cells via secreted cytokines and chemokines, but the contribution of these activities to tumor malignancy requires further investigation. Further studies are also required to decipher the involvement of canonical or non-canonical Notch signaling in this process. Moreover, TME in different types of tumors may respond to SM22-MCs in heterogenous manners, because vessel density showed a tendency of decrease in LLC (Fig. 4a) but increased in B16 tumors (Additional file 1: Figure S4E), although in both cases mural cell coverage of vessels decreased upon RBP-J ablation. More experiments are required to address this question.

The Notch signaling pathway participates in regulating TME development and remodeling, but the consequence of Notch activation is cell type- and/or context-dependent [39]. In tumor stromal cells, loss of RBPj has been shown to promote fibroblast activation and conversion into CAFs and ensures keratinocyte-derived tumors [17, 40]. Notch is also a well-recognized regulator of EMT, thus, influences the generation of cancer cell-derived tumor stromal cells [41]. In vSMCs, Notch signaling plays a critical role in regulating development and plasticity [15]. It is noteworthy that Notch3 is one of the most important regulator of vSMC in different districts [42, 43], and it will be interesting to examine the differential role of each Notch receptor in future. Taking SM22α as a tentative vSMC marker, our results indicate that Notch inhibition promotes the transition of vSMCs from the contractile to secretory phenotype, leading to destabilized microvessels and vicious paracrine factors that facilitate tumor progression.

The mechanism underlying Notch-mediated regulation could be multiple. In TME, Notch signaling crosstalks with many other signals, including TGF-β, WNT, YAP, among others [39]. These interactions form a robust network to elicit a pro-tumor stromal environment via promoting tumor growth and inhibiting anti-tumor immunity [39]. Notch activation could inhibit vSMC proliferation and transdifferentiation via p27 and SOX9, respectively [44]. Goruppi et al. have shown that autophagy, which is frequently activated in TME, regulates CAFs via Notch/RBPj signaling [45]. Moreover, senescence, which is induced by over-proliferation and DNA damage, has been implicated in CAF activation and function [46]. Recent reports have highlighted the role of Notch/RBPj signaling in the regulation of senescence. Procopio et al. have provided evidence that combined RBPj and p53 downregulation promotes CAF activation [16]. Consistently, Bottoni et al. showed recently that RBPj controls telomere maintenance and genome stability in human dermal fibroblasts [40]. These findings imply that blockade of Notch signaling would promote CAF activation and phenotype via promoting senescence. However, other reports have shown that activation of Notch signaling in cancer cells could also promote senescent phenotypes [47]. More intensive studies, especially at the epigenetic level, could be important to reveal the mechanisms underlying Notch-mediated regulation of different subsets of tumor stromal cells.

## Conclusions

SM22α^+^ mural cells facilitate vessel stability in tumors, but they gain a secretory phenotype and promote tumor malignancy in the absence of Notch signaling. Notch activation can repress the secretory phenotype of SM22α^+^ mural cells, leading to normalization of tumor vasculature.

## Supplementary information

**Additional file 1: Figure S1.** Animal models.** Figure S2.** Deletion of SM22-MCs reduced B16 tumor growth.** Figure S3.** Expression of Notch-related genes in vSMCs.** Figure S4.** RBPj deficiency in SM22-MCs promotes B16 tumor progression.** Figure S5.** Tumor cell-derived CM subverts vSMC phenotypes. **Figure S6.** Analyses of AdNIC- or AdCtrl-transduced vSMCs-DA transcriptomes. **Figure S7.** Bioinformatic analyses.

**Additional file 2: Table S1.** Information of patients involved in this study. **Table S2.** Primers used for qRT-PCR and genotyping.** Table S3.** Antibodies used in this study.

## Data Availability

The RNA-seq datasets have been deposited in the BIG Sub database (https://bigd.big.ac.cn/) with an accession # PRJCA002745. Other materials are available from the corresponding authors on request.

## Abbreviations

CAF: Cancer-associated fibroblast
CM: Conditional medium
CTCs: Circulating tumor cells
DTA: Diphtheria toxin A chain
EdU: 5-ethynyl-2′-deoxyuridine
EMT: Epithelial-to-mesenchymal transition
FACS: Fluorescence activated cell sorting
GSEA: Gene set enrichment analysis
H&E: Hematoxylin-eosin staining
LLC: Lewis lung cancer
NF-kB: Nuclear factor kappa B
PBS: Phosphate-buffered saline
PFA: Paraformaldehyde
PIMO: Pimonidazole hydrochloride
qRT-PCR: quantitative real-time polymerase chain reaction
RBPj: Recombination signal binding protein for immunoglobulin kappa J
SM22-MCs: SM22α^+^ mural cells
SFM: Serum-free medium
TCM: Tumor-conditioned medium
TME: Tumor microenvironment
vMCs: vascular mural cells
vSMCs: vascular smooth muscle cells
vSMCs-DA: vSMCs from mouse dorsal aorta
