# Supplementary material for: SM22α+ vascular mural cells are essential for vessel stability in tumors and undergo phenotype transition regulated by Notch signaling
Source: J Exp Clin Cancer Res. 2020 Jul 2;39:124. doi: 10.1186/s13046-020-01630-x (PMC7331127; doi:10.1186/s13046-020-01630-x)
Supplement: Supplementary file 1 — Additional file 1: Figure S1. Animal models. Figure S2. Deletion of SM22-MCs reduced B16 tumor growth. Figure S3. Expression of Notch-related genes in vSMCs. Figure S4. RBPj deficiency in SM22-MCs promotes B16 tumor progression. Figure S5. Tumor cell-derived CM subverts vSMC phenotypes. Figure S6. Analyses of AdNIC- or AdCtrl-transduced vSMCs-DA transcriptomes. Figure S7. Bioinformatic analyses. [file 13046_2020_1630_MOESM1_ESM.docx]

**Additional file 1**

**
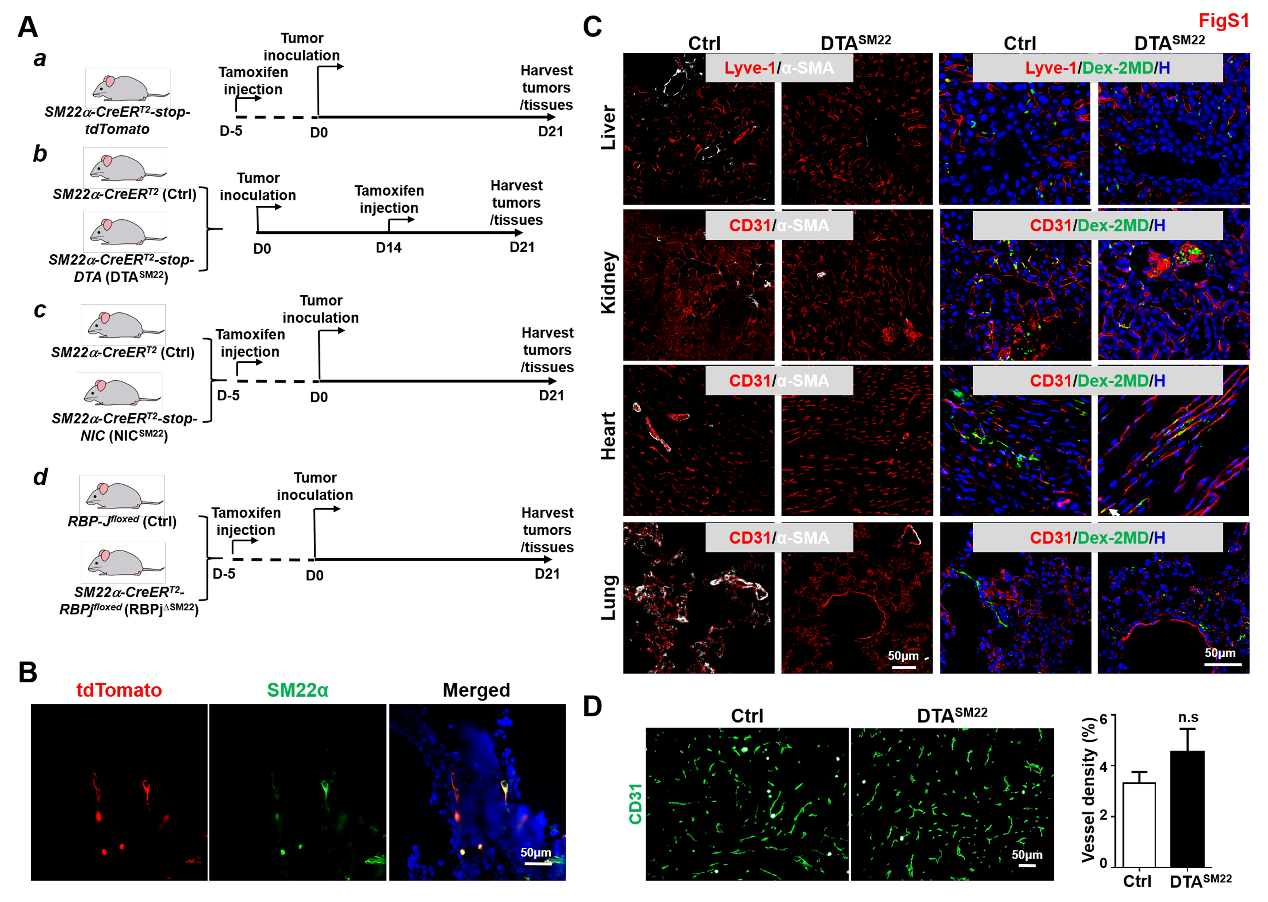
**

Figure S1. Animal models. (**A**) Mice and experimental design. For SM22α-CreER^T2^-stop-tdTomato (tdTomato^SM22^) (*a*), SM22α-CreER^T2^-stop-NIC (NIC^SM22^) (*c*), and SM22α-CreER^T2^-RBPJ^floxed^ (RBPj^∆SM22^) (*d*), mice were daily injected with tamoxifen for 5 consecutive days. LLC or B16 cells were inoculated subcutaneously on mice a day after the last injection. Tumors were collected 21 days after the inoculation. For SM22α-CreER^T2^-stop-DTA (DTA^SM22^) mice (*b*), tumors were inoculated and tamoxifen injection was started from 8th day before tumor recovery. (**B**) LLC tumors from tdTomato^SM22^ mice were stained with anti-SM22α. (**C**) DTA^SM22^ mice were injected with tamoxifen to induce DTA expression. Mice were injected with Dextran-2MD 15 min before being sacrificed. Tissue sections of liver, kidney, heart, and lung were stained by immunofluorescence. (**D**) Tumor sections from DTA^SM22^ mice were stained with CD31, and vessel density was quantitatively compared (n = 5). Bars = means ± SD. n.s, not significant.

**
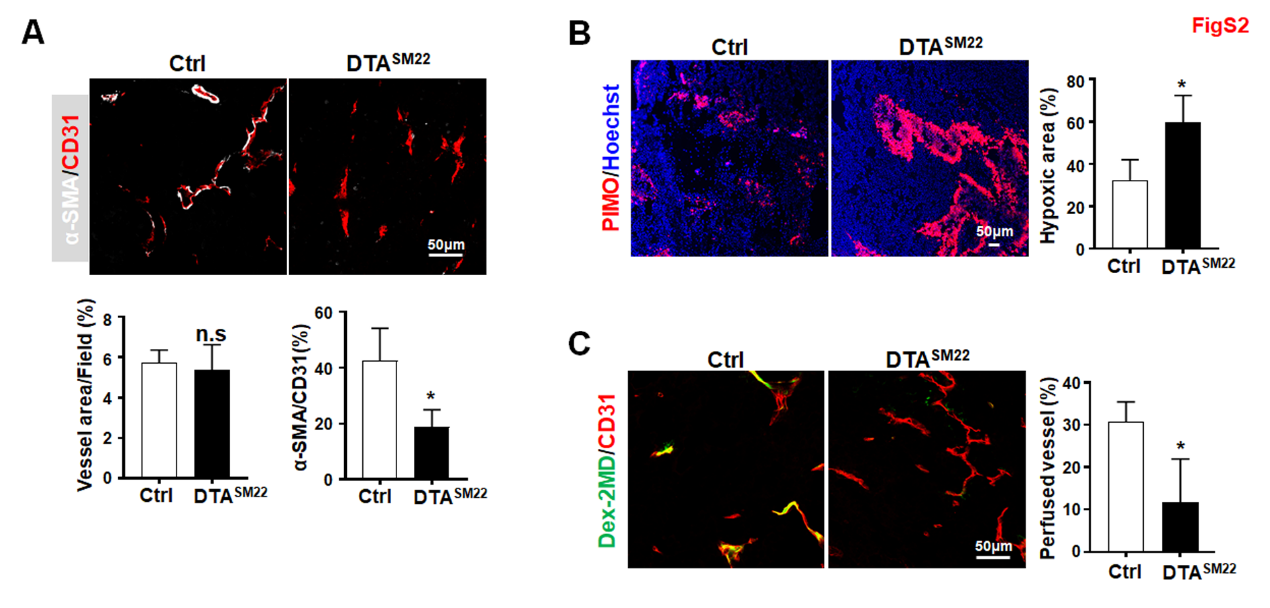
**

Figure S2. Deletion of SM22-MCs reduced B16 tumor growth. (**A**) B16 cells were inoculated in DTA^SM22^ and Ctrl mice. Tumor sections were coimmunostained with CD31 and α-SMA. The percentage of CD31/field and α-SMA/CD31 was quantitatively compared. (**B**) Tumor sections from (A) were stained with PIMO. Hypoxic areas were quantitatively compared. (**C**) Tumor-bearing mice were injected i.v with FITC-Dextran-2MD 15 min before sacrificed. Tumor sections were immunostained with CD31. Perfused vessels (CD31^+^Dex-2MD^+^) were quantitatively compared between the DTA^SM22^ and Ctrl mice (n = 3). Bars = means ± SD. *, P < 0.05; n.s, not significant.

**
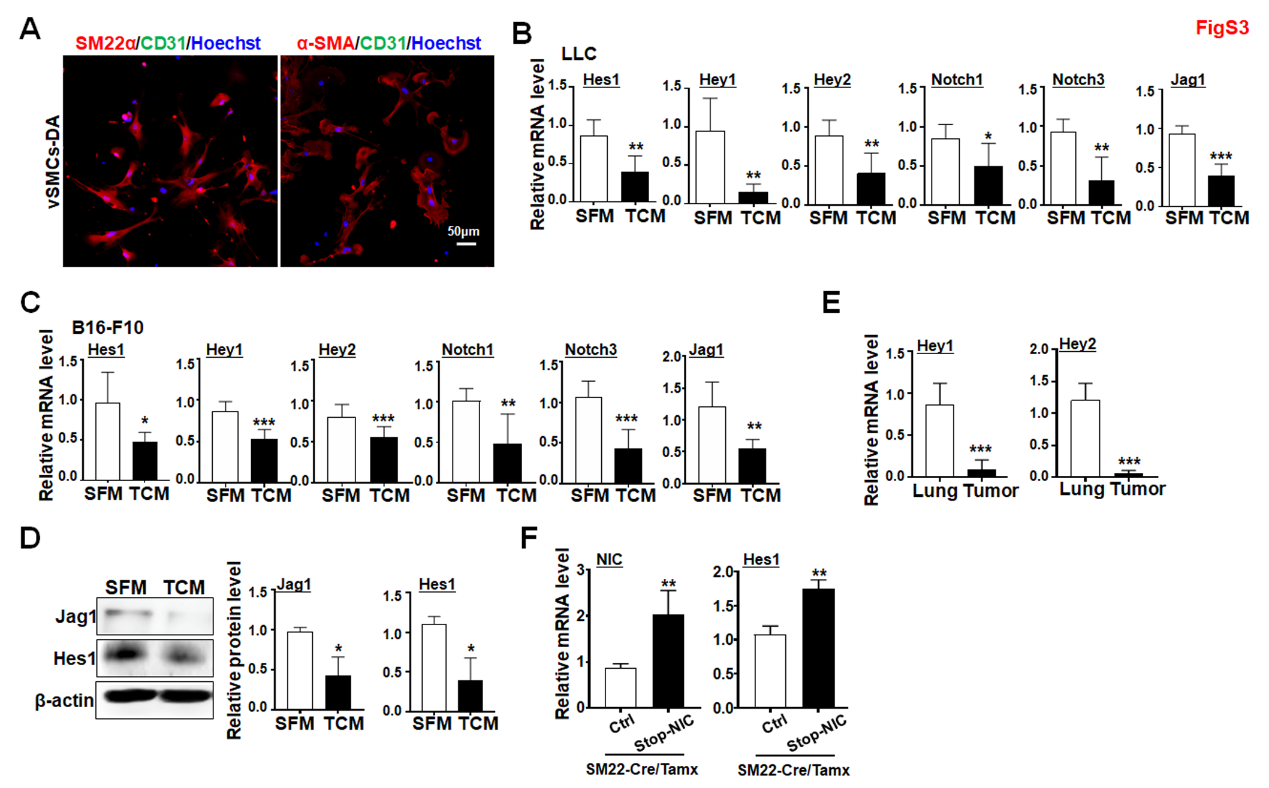
**

Figure S3. Expression of Notch-related genes in vSMAs. (**A**) vSMCs-DA were isolated from wild type mice and stained by immunofluorescence with anti-SM22α, anti-α-SMA, and anti-CD31. (**B**, **C**) vSMCs-DA were cultured with LLC- (A) or B16-derived (B) CM (TCM) for 48 h, with serum-free medium (SFM) as a control. The mRNA level of Notch-related genes was determined by qRT-PCR (n = 5 for LLC; n = 4 for B16). (**D**) vSMCs-DA were stimulated as in (B) and Jag1 and Hes1 protein levels were evaluated by western blotting, with β-actin as an internal control (n = 3). (**E**) SM22-MCs were isolated from LLC tumors or adjacent lung tissues from tdTomato^SM22^ mice by FACS-sorting, and Hey1 and Hey2 mRNA levels were determined by qRT-PCR (n = 6). (**F**) vSMCs-DA were isolated from Ctrl and NIC^SM22^ mice. mRNA level of NIC and Hes1 was determined by qRT-PCR (n = 3). Bars = means ± SD. *, P < 0.05; **, P < 0.01; ***, P < 0.001.


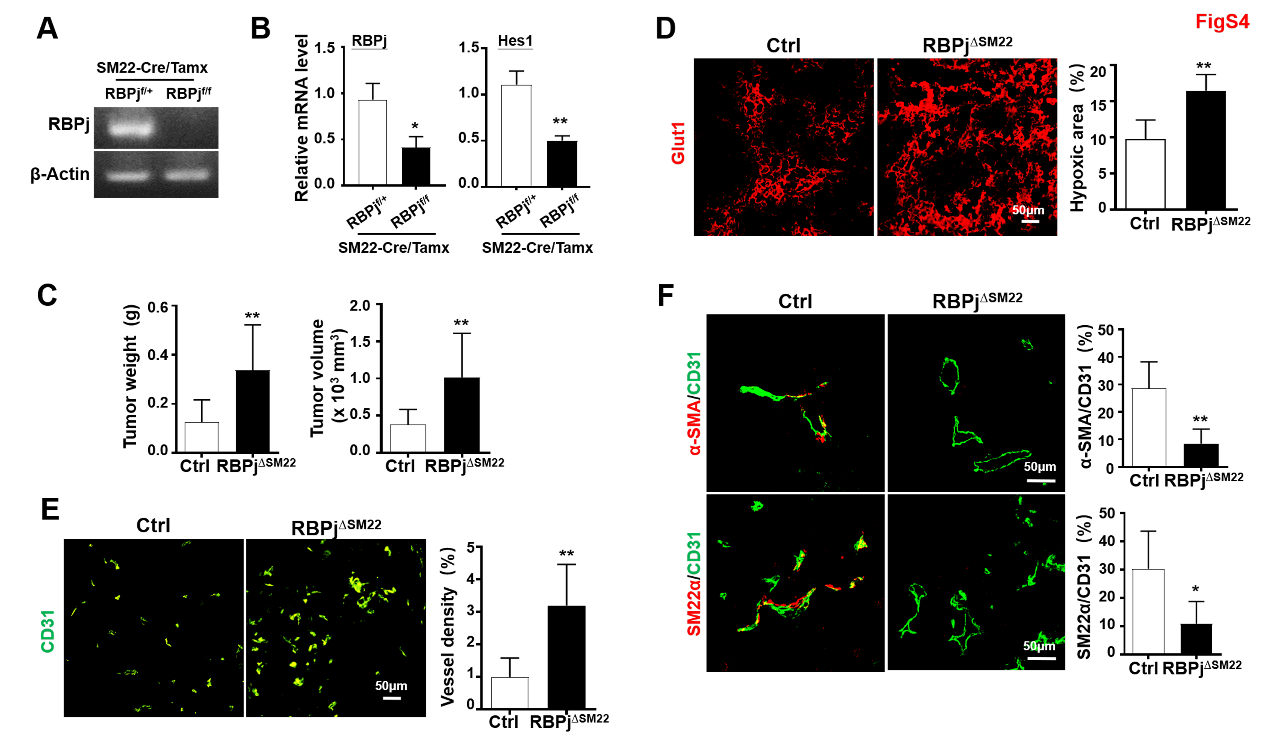


Figure S4. RBPj deficiency in SM22-MCs promotes B16 tumor progression. (**A**, **B**) vSMCs-DA were isolated from Ctrl and RBPj^∆SM22^ mice. Deletion of the floxed exon of RBPj was determined by PCR (A). The mRNA levels of RBPj and Hes1 were determined by qRT-PCR (n = 3). (**C**) B16-F10 tumor cells were inoculated in RBPj^∆SM22^ and Ctrl mice for 16 days. Tumors were harvested and tumor weight and volume measured and quantitatively compared (n = 10 for Ctrl; n = 9 for RBPj^∆SM22^). (**D**) Tumor sections from (A) were immunostained with Glut1 and the percentage Glut1^+^ areas were quantitatively compared (n = 5). (**E**) Tumor vessel density in (A) was evaluated by CD31immunostaining and quantitatively compared (n = 5). (**F**) Tumor sections from (A) were immunostained with CD31 plus α-SMA or SM22α, and the α-SMA/CD31 and SM22α/CD31 percentages determined (n = 5). Bars = means ± SD. *, P < 0.05; and **, P < 0.01.


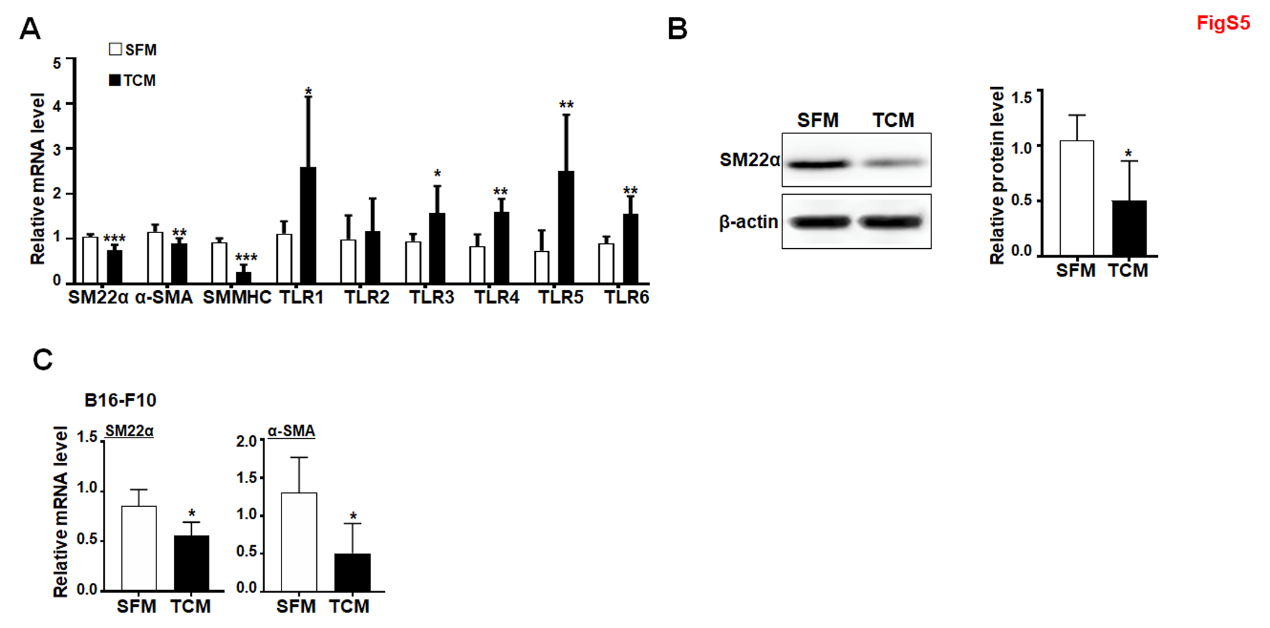


**Figure S5.** Tumor cell-derived CM subverts vSMC phenotypes. (**A**) vSMCs-DA were cultured with LLC-derived CM (TCM) for 48 h, with serum-free medium (SFM) as a control. The mRNA levels of SM22α, α-SMA, SMMHC, and TLR1-6 were determined using qRT-PCR (n = 5). (**B**) vSMCs-DA were stimulated as in (A) and the SM22α protein level was evaluated by western blotting (n = 3). (**C**) vSMCs-DA were cultured with B16-derived CM for 48 h. SM22α and α-SMA mRNA levels were determined using qRT-PCR (n = 4). Bars = means ± SD. *, P < 0.05; **, P < 0.01; ***, P < 0.001.

**
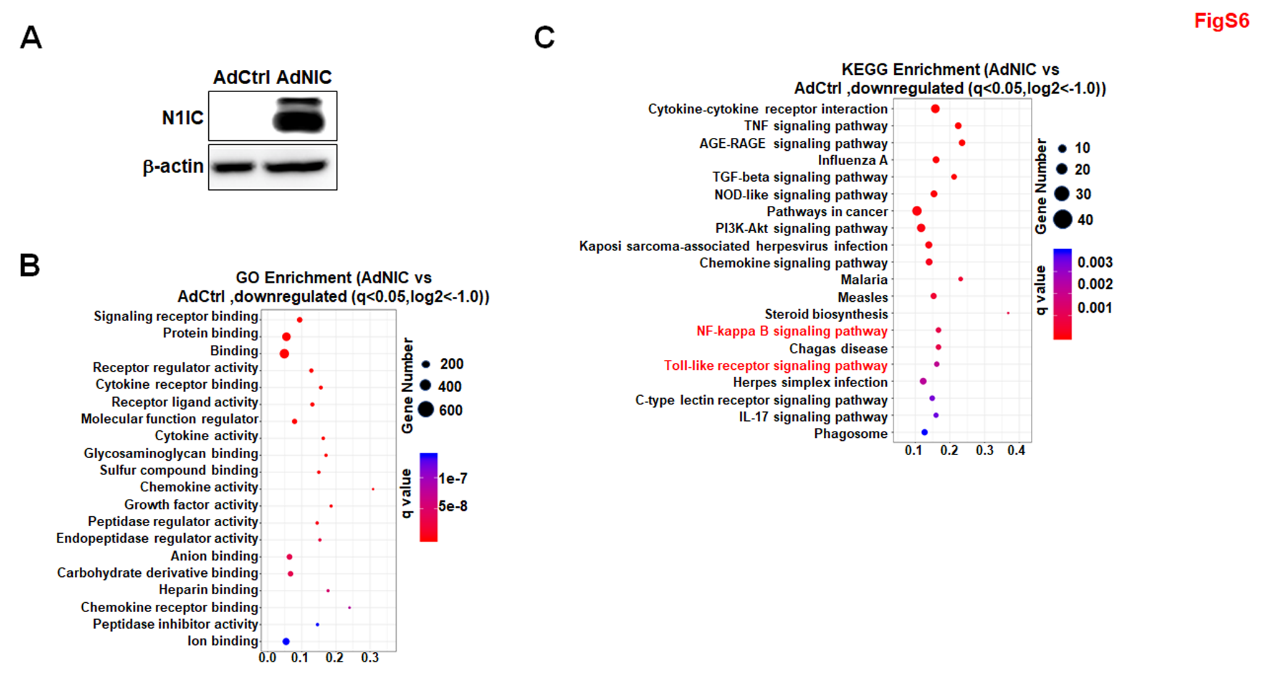
**

Figure S6. Analyses of AdNIC- or AdCtrl-transduced vSMCs-DA transcriptomes**.** (**A**) vSMCs-DA were transduced with AdNIC or AdCtrl, and the expression of Notch1 NIC (N1IC) was determined by western blotting. (**B**) vSMCs-DA were transduced with AdNIC or AdCtrl and subjected to RNA-seq. The differentially downregulated genes (q < 0.05 and log2 < -0.1) in the AdNIC group were analyzed using edgeR analysis and then subjected to a GO enrichment analysis. The picture shows the top 20 significantly changed entries. (**C**) The differentially downregulated genes as in (A) were subjected to KEGG enrichment analysis. The picture shows the top 20 significantly changed entries, in which the TLR and NF-κB signaling pathways were marked in red.


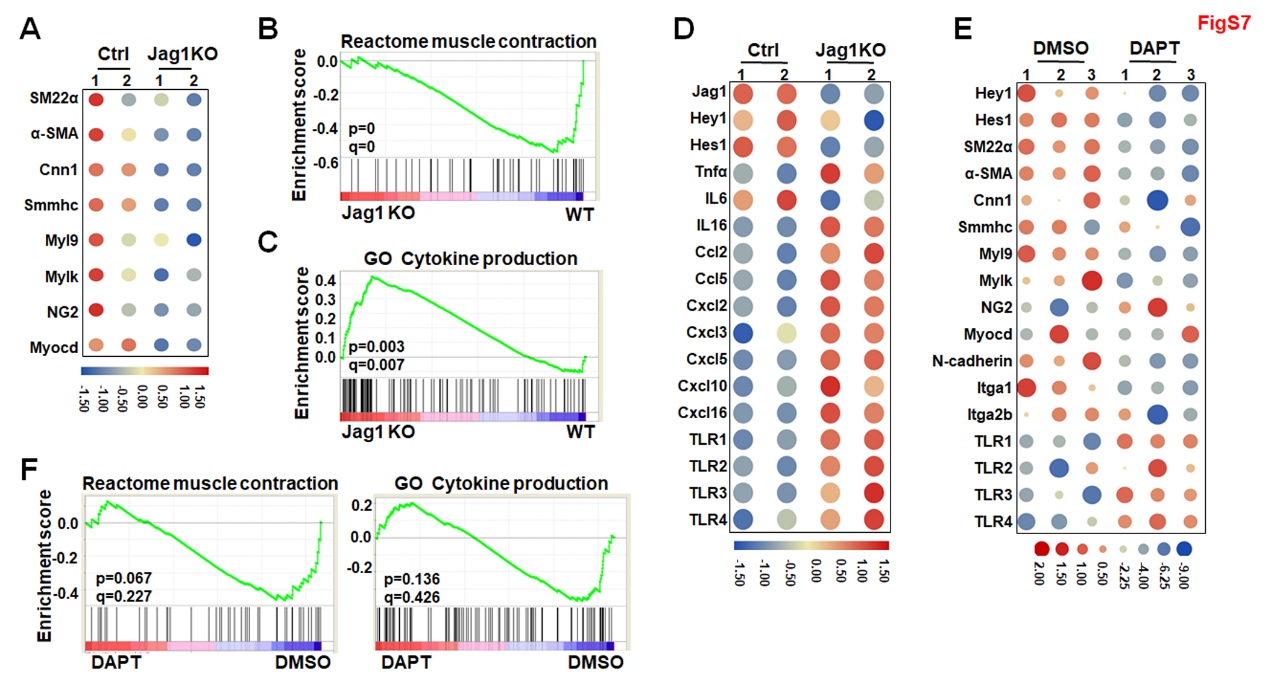


Figure S7. Bioinformatic analyses**.** (**A**-**D**) Jag1 KO and control vSMCs (GSE60643) transcriptomes were analyzed by heatmap or GSEA to show the differential expression of contractile (A, B) and secretory (C, D) phenotype-related genes. (**E**, **F**) The expression of contractile and secretory phenotype-related genes was compared using a heatmap (E) and GSEA (F) in DAPT- and DMSO-treated vSMCs (GSE60643).
