# Supplementary material for: SM22α+ vascular mural cells are essential for vessel stability in tumors and undergo phenotype transition regulated by Notch signaling
Source: J Exp Clin Cancer Res. 2020 Jul 2;39:124. doi: 10.1186/s13046-020-01630-x (PMC7331127; doi:10.1186/s13046-020-01630-x)
Supplement: Supplementary file 2 — Additional file 2: Table S1. Information of patients involved in this study. Table S2. Primers used for qRT-PCR and genotyping. Table S3. Antibodies used in this study. [file 13046_2020_1630_MOESM2_ESM.docx]

**Additional file 2**

Table S1. Information of patients involved in this study.

| **No.** | **Sex*** | **Age**  **(years)** | **Pathological classification** | **TNM stage** | **Pathological**  **grade** | **Clinical stage** |
| --- | --- | --- | --- | --- | --- | --- |
| 1 | F | 58 | adenocarcinoma | T3N1M0 | unclear | unclear |
| 2 | M | 77 | adenocarcinoma | T3N0M0 | low differentiation | IV |
| 3 | F | 68 | adenocarcinoma | T2N0M0 | low differentiation | IIb |
| 4 | M | 68 | adenocarcinoma | T3N1M0 | moderate differentiation | IIIa |

*M, male; F, female.

Table S2. Primers used for qRT-PCR and genotyping.

| **Gene** | **Forward (5’-3’)** | **Reverse (5’-3’)** |
| --- | --- | --- |
| Mouse Notch1 | GATGGCCTCAATGGGTACAAG | TCGTTGTTGTTGATGTCACAGT |
| Mouse Notch3 | TGCCAGAGTTCAGTGGTGG | CCATTTTCGCAGGGATGAGAT |
| Mouse Hes1 | TCAACACGACACCGGACAAAC | ATGCCGGGAGCTATCTTTCTT |
| Mouse Hey1 | CCGACGAGACCGAATCAATAAC | TCAGGTGATCCACAGTCATCTG |
| Mouse Hey2 | AAGGCTGGGTGAAGACCCTTA | TGAATGGCCGTTTCTGGAAGT |
| Mouse Jag1 | AATCGCATCGTACTGCCTTTC | GTGTCATTACTGGAATCCCAGG |
| Mouse TNFα | CCCTCACACTCAGATCATCTTCT | GCTACGACGTGGGCTACAG |
| Mouse Ccl2 | TTAAAAACCTGGATCGGAACCAA | GCATTAGCTTCAGATTTACGGGT |
| Mouse Cxcl10 | CCAAGTGCTGCCGTCATTTTC | GGCTCGCAGGGATGATTTCAA |
| Mouse TLR1 | TGAGGGTCCTGATAATGTCCTAC | AGAGGTCCAAATGCTTGAGGC |
| Mouse TLR2 | GCAAACGCTGTTCTGCTCAG | AGGCGTCTCCCTCTATTGTATT |
| Mouse TLR3 | GTGAGATACAACGTAGCTGACTG | TCCTGCATCCAAGATAGCAAGT |
| Mouse TLR4 | ATGGCATGGCTTACACCACC | GAGGCCAATTTTGTCTCCACA |
| Mouse SM22α | CAACAAGGGTCCATCCTACGG | ATCTGGGCGGCCTACATCA |
| Mouse α-SMA | GTCCCAGACATCAGGGAGTAA | TCGGATACTTCAGCGTCAGGA |
| Mouse β-actin | GGCTGTATTCCCCTCCATCG | CCAGTTGGTAACAATGCCATGT |
| Mouse Smmhc | AAGCTGCGGCTAGAGGTCA | CCCTCCCTTTGATGGCTGAG |
| Mouse RBPj | CACTGTTCAATCGCCTTC | AGTCACTGAGCACACAAG |
| Mouse RBPj genome | GCGTGCCTCCCCGCATCTA | CGTTCCTGAAGCAATGCACACTG |
| Mouse β-actin genome | CTCAGTAACAGTCCGCCTAGAA | GAGAGCTCACCATTCACCATCT |
| Mouse NIC | AAGTGGACATTGACGAGTG | AGGCATAAGCAGAGGTAGG |
| Cre | CCGGTCGATGCAACGAGTGATGAGG | GCCTCCAGCTTGCATGATCTCCGG |
| DTA | ACTAAAAGTGGATAATGCCGAAA | CTTCACAAAGAGATCGCCTGACA |
| N1 common | AAAGTCGCTCTGAGTTGTTAT | |
| N1 wild type | TAAGCCTGCCCAGAAGACTC | |
| N1 mutant | GAAAGACCGCGAAGAGTTTG | |
| R3 | GTTCTTAACCTGTTGGTCGGAACC | |
| R4 | GCTTGAGGCTTGATGTTCTGTATTGC | |
| PGKD | ACCGGTGGATGTGGAATGTGT | |
| oIMR9020 | AAGGGAGCTGCAGTGGAGTA | |
| oIMR9021 | CCGAAAATCTGTGGGAAGTC | |
| oIMR9103 | GGCATTAAAGCAGCGTATCC | |
| oIMR9105 | CTGTTCCTGTACGGCATGG | |

Table S3. Antibodies used in this study.

| **Product name** | **Supplier** | **Product code** |
| --- | --- | --- |
| Anti-CD31 | BD Pharmingen | 550274 |
| Anti-CD31-FITC | Invitrogen | 11-0319-42 |
| Anti-CD45-APC | Invitrogen | MHCD4505 |
| Anti-SM22α | Abcam | Ab14106 |
| Anti-α-SMA | Abcam | Ab124964 |
| Anti-GLUT1 | Abcam | Ab15309 |
| Anti-SMMHC | Abcam | Ab53219 |
| Anti-LYVE-1 | Relia Tech | 103-M130 |
| Anti-CNN1 | Abcam | Ab46794 |
| Anti-Ki67 | Millipore | AB9260 |
| Anti-phospho-IkBα | CST | 2859s |
| Anti-IκBα | CST | 4814s |
| Anti-p65 | CST | 8242s |
| Anti-LaminA/C | CST | 4777s |
| Anti-HES1 | CST | 11988s |
| Anti-NIC | Sigma | SAB4502019 |
| Anti-JAG1 | CST | 70109s |
| Anti-β-Actin | CST | 3700s |
| HRP-anti-mouse IgG | CST | 7076s |
| HRP-anti-rabbit IgG | CST | 7074s |
| Alexa Fluor® 488-anti-rat IgG | Invitrogen | A-21208 |
| Alexa Fluor®594-anti-rat IgG | Invitrogen | A-11007 |
| Alexa Fluor®647-anti-rat IgG | Invitrogen | A-21245 |
| Alexa Fluor®594-anti-rabbit IgG | Jackson | 711-586-152 |
| Alexa Fluor®488-anti-rabbit IgG | Jackson | 711-545-152 |
